# Pharmacokinetics and Metabolism of Naringin and Active Metabolite Naringenin in Rats, Dogs, Humans, and the Differences Between Species

**DOI:** 10.3389/fphar.2020.00364

**Published:** 2020-03-27

**Authors:** Yang Bai, Wei Peng, Cuiping Yang, Wei Zou, Menghua Liu, Hao Wu, Loudi Fan, Peibo Li, Xuan Zeng, Weiwei Su

**Affiliations:** ^1^Guangdong Engineering and Technology Research Center for Quality and Efficacy Re-evaluation of Post-Marketed Traditional Chinese Medicine, School of Life Sciences, Sun Yat-sen University, Guangzhou, China; ^2^Guangdong Key Laboratory of Plant Resources, School of Life Sciences, Sun Yat-sen University, Guangzhou, China

**Keywords:** pharmacokinetics, metabolism, rat, dog, human, naringin, naringenin, species differences

## Abstract

**Background:**

Pharmacokinetics provides a scientific basis for drug product design, dosage regimen planning, understanding the body's action on the drug, and relating the time course of the drug in the body to its pharmacodynamics and/or toxic effects. Recently, naringin, a natural flavonoid, was approved for clinical trials as a first-class new drug product by the China Food and Drug Administration, owing to its nonclinical efficacy in relieving cough, reducing sputum, and low toxicity. Previous reports focused on the pharmacokinetic studies of naringin or its active metabolite naringenin in rats, which were scattered and insufficient because naringin was coadministered with mixtures such as herbs, fruits, and other traditional medicines. The purpose of this study was to evaluate the pharmacokinetics and metabolism of naringin and naringenin, following oral and intravenous administration of naringin in rats, dogs, and humans, which can be beneficial for new drug development.

**Methods:**

Separate bioanalytical methods were developed and validated to determine the concentrations of naringin and its active metabolite naringenin in biological samples obtained from rats, dogs, and humans. Comprehensive nonclinical and clinical data were used to estimate the pharmacokinetic parameters of naringin and naringenin. Experiments included single-dose studies (oral and intravenous administration), multiple-dose studies, and an assessment of food-effects. Furthermore, the metabolism of naringin and naringenin was studied in rat and human liver and kidney microsomes. All biological samples were analyzed using liquid chromatography-tandem mass spectrometry.

**Results:**

The pharmacokinetic parameters of naringin and naringenin were calculated and the results show an insignificant influence of high-fat diet and insignificant accumulation of the drugs after multiple dosing. Twelve metabolites were detected in the liver and kidney microsomes of rats and humans; naringin metabolism was a complex process simultaneously catalyzed by multiple human enzymes. All evaluated species demonstrated differences in the pharmacokinetics and metabolism of naringin and naringenin.

**Conclusion:**

The results can be used to design a dosage regimen, deepen understanding of mechanisms, and accelerate new drug development.

**Clinical Trial Registration:**

http://www.chinadrugtrials.org.cn/eap/main, identifiers CTR20130704 and CTR20190127.

## Introduction

Pharmacokinetics is the science of the kinetic processes of drug absorption and disposition (i.e., distribution, metabolism, and excretion), which can provide a scientific basis for drug product design, dosage regimen design, understanding the body's action on the drug, and relating time course of the drug in the body to its pharmacodynamics and toxic effects.

In the last 2 decades, extensive research has been devoted to developing naringin as a first-class new drug to relieve cough and reduce sputum, and the drug has been approved for clinical trials by the China Food and Drug Administration (Li et al., 2015). In pharmacological studies, naringin exhibited antitussive and protective effects against acute lung injury, pulmonary fibrosis, chronic bronchitis, and cough-variant asthma (Gao et al., 2011; Liu Y. et al., 2011; Liu Y. et al., 2012; Luo et al., 2012; Nie et al., 2012a; Nie et al., 2012b; Chen et al., 2013; Luo et al., 2013; Chen et al., 2014; Jiao et al., 2015). Furthermore, naringin toxicity was assessed in Sprague-Dawley rats (Liu M. et al., 2011; Li P. et al., 2013; Li et al., 2014). In a study assessing a single p.o. bolus naringin dose (16 g kg^-1^), no apparent toxicity was documented (Li P. et al., 2013). Additionally, no adverse effects were observed in a toxicity study of naringin (1,250 mg kg^-1^ day^-1^ for 184 days) (Liu M. et al., 2011; Li et al., 2014).

With the exception of methods available in the literature on the analysis of rat plasma (Tsai and Tsai, 2012; Wen et al., 2012; Tong et al., 2012; Li S. Q. et al., 2013; Jin et al., 2015; Lu et al., 2015; Zeng et al., 2018; Zeng et al., 2019) and human plasma and urine (Ishii et al., 2000; Xiong et al., 2014), only a few methods are reported to analyze naringin and naringenin in other types of biological matrices in rats, dogs, and humans. The present study reports the analysis of naringin and naringenin in rat, dog, and human plasma and human urine and feces. Previously, the pharmacokinetic studies of naringin or naringenin focused on rats, and naringin was often coadministered in mixtures such as herbs, fruits, and other traditional medicines (Tsai and Tsai, 2012; Tong et al., 2012; Wen et al., 2012; Li S. Q. et al., 2013; Jin et al., 2015; Lu et al., 2015). Hence, in rats, the pharmacokinetics of naringin could be influenced by other coadministered constituents. Moreover, naringin pharmacokinetics has been minimally investigated in dogs and humans. Hence, these pharmacokinetic parameters are inaccurate and insufficient to design drug dosage regimens in clinical trials. In the current study, the pharmacokinetic parameters of naringin and naringenin were estimated accurately and sufficiently, since naringin was administered alone to rats, dogs, and humans. Furthermore, the metabolic processes of naringin were studied using human excreta. Following oral administration, naringin is metabolised to the active metabolite naringenin and their hydroxylated, hydrogenated, dehydrogenated, and acetylated metabolites by intestinal microflora (Chen et al., 2018). Subsequently, the metabolites are transported, mainly *via* the portal vein, to the liver for further biotransformation to glucuronide and sulphate conjugates, which enter systemic circulation (Tsai and Tsai, 2012). Finally, naringin, naringenin, and their conjugates are secreted into bile and excreted into the feces or reabsorbed to become systemically available (i.e., through enterohepatic circulation) (Ma et al., 2006; Liu M. et al., 2012; Zeng et al., 2017). Since the glucuronide and sulphate conjugates of flavonoids were reported as precursors of bioactive metabolites (glycoside or aglycone) (Terao et al., 2011; Matsumoto et al., 2018), hydrolysis with β-glucuronidase is often used in pharmacokinetic studies of naringin or naringenin. Although the metabolism and nonclinical excretion of naringin have previously been reported, enzyme pathways and excretion were insufficiently presented in humans, hindering elucidation of the clinical mechanisms (Liu M. et al., 2012; Zeng et al., 2017). Our study focused on the metabolism of naringin with regards to enzyme pathways and its excretion in humans, which is beneficial to comprehend the mechanisms of naringin in clinical studies.

In this study, we investigated the pharmacokinetics and metabolism of naringin and naringenin in rats, dogs, and humans, following the administration of naringin. Separate bioanalytical methods were employed for the analysis of naringin and naringenin in biological samples obtained from rats, dogs, and humans. An abundance of data was utilized to calculate the pharmacokinetic parameters of naringin and naringenin in rats, dogs, and humans, including naringin administration as a single dose (oral, p.o.; intravenous, i.v.), in multiple doses, and with a high-fat diet. Moreover, the metabolism of naringin and naringenin was researched in rat and human liver and kidney microsomes, indicating small species differences in metabolites, with a complex metabolic process simultaneously catalyzed by multiple human enzymes. Species differences were observed with regards to the pharmacokinetics and metabolism of naringin. This information contributes to naringin development as a new drug and understanding the *in vivo* mechanisms involved.

## Materials and Methods

### Materials

Standard naringin (Lot: 110722-201714, purity 94.3%), hesperetin (Lot: 110730-200512, purity 98.0%), hesperidin (Lot: 110721-201617, purity 96.1%), and ketoconazole (Lot: 100294-201404, purity 99.0%) were obtained from the National Institute for the Control of Pharmaceutical and Biological Products (Beijing, China). Standard naringenin (Lot: BCBT8724, purity 97.0%), quercetin 3-β-D-glucoside (Lot: 131697, purity 90.5%), β-glucuronidase from *Helixpomatia* (G0751-100KU, Lot: SLBT2889, ≥300,000 units g^-1^ solid), neohesperetin (Lot: 061K123, purity 90.0%), rhoifolin (Lot: 023H0743, purity 99.0%), neoeriocitrin (Lot: 72129, purity 95.0%), 4-hydroxyphenylpropionic acid (Lot: H52406, purity 98.0%), sulfaphenazole (UC166, purity 98.0%), and quinidine (S7809, purity 98.0%) were purchased from Sigma-Aldrich (St. Louis, MO, USA). Standard α-naphthoflavone (A130000, purity 98.0%), (+/−)-N-3-benzylnirvanol (B285770, purity 98.0%) were purchased from Toronto Research Chemicals (North York, Ontario, Canada). Naringin-D4 (Lot: AC-043-055A1, purity 97.39%) and naringenin-D4 (Lot: AC-043-209A1, purity 99.0%) were purchased from Artis Chemistry Co. Ltd (Shanghai, China). Standard apigenin (Lot: Q023, purity 98.0%) was purchased from Tianjin Jianfeng Natural Products R&D Co. Ltd (Tianjing, China). Standard 5, 7-dihydroxy chromanone (Lot: TH20080915, purity 99.0%) and eriodictyol (Lot: TH20090705, purity 99.0%) were purchased from SINOVA Inc. (Bethesda, Maryland, USA). For nonclinical experiments, naringin was extracted from *Citri grandis* exocarpium, with a purity of 98.8%, which was determined using HPLC with an external standard. Naringin tablets (Lot: 140201), containing 40 mg naringin each, and placebo tablets were manufactured by Guangdong Huanqiu Pharmaceutical Co., Ltd (Fosha, Guangdong, China). Human liver microsomes (10-Donor Pooled) and Sprague-Dawley rat liver and kidney microsomes were purchased from RILD Research Institute for Live Diseases (Shanghai, China) Co.Ltd.

### Animals and Drug Administration

All experimental procedures and protocols were conducted in accordance with the Basel Declaration and National Institutes of Health guide for the care and use of laboratory animals (NIH Publications No. 8023, revised 1978), and approved by the Animal Ethics Committee of the School of Life Sciences at Sun Yat-sen University (Guangzhou, Guangdong, China; Approval number: 090108). All nonclinical experimental designs were consistent with the Technical Guidelines for Nonclinical Pharmacokinetic Studies of Drugs published by the China Food and Drug Administration. Animal studies were reported in compliance with the Animal Research: Reporting *In Vivo* Experiments guidelines.

Sixty healthy adult Sprague Dawley rats (180–220 g, half male and female) were purchased from the Guangdong Medical Laboratory Animal Center (Guangzhou, Guangdong, China). Thirty Beagle dogs (10–14 kg, half male and female) were purchased from the Gaoyao Kangda Laboratory Animal Science & Technology Co., Ltd. (Gaoyao, Guangdong, China). All animals were housed under humanized conditions with free access to food and water, and acclimated to the living conditions for at least one week prior to experimentation at the Laboratory Animal Center of the School of Life Sciences, Sun Yat-sen University. Environmental conditions were maintained at 20°C–25°C, 55% ± 15% relative humidity, 12-h light/dark cycles, and 12 air changes per hour. Prior to experimentation, the animals were fasted overnight with water available *ad libitum*. All animals were healthy based on physical examination and laboratory analyses performed before and after the experiments. At the end of the experiments, the dogs were returned to the Laboratory Animal Center of School of Life Sciences, Sun Yat-sen University.

To comprehensively estimate the pharmacokinetic parameters and absolute bioavailability, rats were randomly divided into six groups and administered naringin (i.v., 42 mg kg^-1^; p.o., 10.5, 21, 42, and 168 mg kg^-1^; p.o., three times from the 1^st^ to 8^th^ day, and once on the 9^th^ day, 42 mg kg^-1^), and dogs were randomly divided into five groups and administered naringin (i.v., 12.4 mg kg^-1^; p.o., 3.1, 12.4, and 49.6 mg kg^-1^; p.o., three times from the 1^st^ to 6^th^ day, and once on the 7^th^ day, 12.4 mg kg^-1^).

### Humans and Drug Administration

Participants were healthy males and females, 18 to 40 years old, ≥50 kg weight, 19 to 25 kg m^2^ body mass index. The health condition of participants was considered good based on the screened medical history, physical examination, vital signs, electrocardiogram, and laboratory examination. A 12-lead electrocardiogram and serum troponin I had to be normal at screening within 8 days of initial dosing, and serology for hepatitis C antibodies, hepatitis B surface antigen, human immunodeficiency virus (HIV) antibodies, syphilis antibodies, HIV, and blood pregnancy tests had to be negative. Clinical laboratory test results (sodium, potassium, total protein, white blood and red blood cell counts, hematocrit, hemoglobin, platelet count, aspartate aminotransferase, alanine aminotransferase, gamma-glutamyl transferase, blood urea nitrogen, creatinine, total bilirubin, alkaline phosphatase, creatine kinase, lactate dehydrogenase, calcium, chloride, phosphorus, albumin, globulin, cholesterol, triglycerides, glucose, uric acid, and urinalysis) had to be within normal ranges, without other clinically significant abnormalities. During the study duration, participants were required to use barrier contraception with spermicide during sexual intercourse. During the trial, female participants were in the nonmenstrual, nonpregnant, and nonlactation periods, and they were nonsmokers. Participants were not planning a pregnancy within 6 months of the clinical trials, they communicated with the investigator, understood and complied with the study requirements, and signed informed consent.

The main exclusion criteria were history of a clinically significant disease, an abnormality, or drug allergies, including a history or evidence of cardiovascular, respiratory, hepatic, renal, haematologic, gastrointestinal, endocrine, metabolic, immunologic, dermatologic, neurologic, oncologic, or psychiatric illness or significant abnormalities, or any condition, surgical intervention known to interfere with drug absorption, distribution, metabolism, or excretion. Prior to clinical trials, participants were excluded if they had acute or subacute cough, mediated by neurogenic inflammation (such as cough after cold), chronic bronchitis with chronic cough, bronchial asthma, chronic obstructive pulmonary disease, pulmonary cystic fibrosis, bronchiectasis, and other respiratory diseases. Participants were excluded if they had abnormal chest radiographs or electrocardiogram parameters as follows: PR intervals >210 ms, or QRS duration >120 ms, or QTcB >460 ms (male) or QTcB >470 ms (female). In addition, participants were excluded if they planned to have a baby within 6 months posttrials. A history of significant tea or coffee consumption (> 8 cups per day) was prohibited. Participants were excluded if they had been administered with any investigational products in clinical studies four weeks prior to this trial or any other drugs, herbal products, and dietary supplements two weeks before this trial.

Briefly, the study was a randomized, double-blinded, and placebo-controlled phase I clinical trial, including a single ascending dose and multiple-dose studies of naringin tablets administered to seven groups of healthy adult subjects (10 subjects in each group, 2 received placebo). Naringin and placebo tablets were identical in appearance and participants, care providers, and those assessing outcomes were blinded to the study drug assignment. Using the Statistical Analysis System (SAS) software (Version 9.4, SAS Institute Inc., Cary, NC, USA), subject randomization and blinding/masking were performed by statisticians from the Department of Health Statistics at the Second Military Medical University. Naringin tablets were administered p.o. in single doses of 40, 80, 160, 160 (high-fat diet), 320, and 480 mg, separately, and in multiple doses of 160 mg (once on the 1^st^ day, three times from 2^nd^ to 6^th^ day, and once on the 7^th^ day).

Clinical studies (registration numbers: CTR20130704 for single-dose studies and CTR20190127 for multiple-dose study, http://www.chinadrugtrials.org.cn/eap/main) were conducted, in accordance with the International Conference on Harmonization (ICH) E6 Consolidated Guidance for Good Clinical Practice (GCP) (1996) and the US Code of Federal Regulations (CFR) 21 parts 50 and 56. The clinical study was conducted at the Beijing Hospital; the protocols and informed consent forms were approved by the Institutional Review Board at the Beijing Hospital (Beijing, China; Approval number: 2014BJYYEC-047-14; 2018BJYYEC-123-02) and participants provided written informed consent before any study-related procedures were performed, in accordance with the Helsinki Declaration (1996). Data administration was conducted by the Department of Health Statistics at the Second Military Medical University, in accordance with ICH of Technical Requirements for Pharmaceuticals for Human Use, GCP, FDA 21 CFR Part 11, and corresponding regulations.

### Pharmacokinetics

Rat blood samples (0.5 ml) were drawn from the orbital veins at 0, 0.25, 0.5, 0.75, 1, 2, 3, 4, 6, 8, 12, 24, and 36 h for the p.o. groups; 0.03, 0.08, 0.25, 0.5, 1, 2, 3, 6, 8, 12, 24, and 36 h for the i.v. group; and 24, 48, 72, 96, 120, 144, 168, 192, 192.25, 192.75, 193, 194, 195, 196, 198, 200, 204, and 216 h for the multiple-dose group. Dog blood samples (1 ml) from a suitable antecubital vein were collected at 0, 0.5, 1, 1.5, 2, 3, 4, 6, 8, 10, 12, 24, and 36 h for the p.o. groups; 0, 0.08, 0.25, 0.5, 0.75, 1, 2, 4, 6, 8, 10, 12, and 24 h for the i.v. group; and 24, 48, 72, 96, 120, 144, 144.5, 145, 145.5, 146, 147, 148, 150, 152, 154, 156, 168, and 180 h for the multiple-dose group.

Human blood samples (4 ml) were collected at 0, 0.25, 0.5, 0.75, 1, 2, 3, 4, 5, 6, 7, 8, 10, 12, 14, 16, 24, 36, and 48 h for the single-dose groups, and 0, 0.25, 0.5, 0.75, 1, 2, 3, 4, 5, 6, 7, 8, 10, 12, 14, 16, 24, 120, 128, 136, 144, 144.25, 144.75, 145, 146, 147, 148, 149, 150, 151, 152, 154, 156, 158, 160, and 168 h for the multiple-dose group. Human urine samples were collected at −12~0, 0~4, 4~8, 8~12, 12~24, 24~36, 36~48, 48~60, and 60~72 h for the single-dose groups and −12~0 (Day1), −12~0 (Day7), 0~4, 4~8, 8~12, 12~24, 24~36, 36~48, 48~60, and 60~72 h (Day7) for the multiple-dose group with records of volume. Human feces were collected in storage boxes, weighed, and recorded from 12 h before administration to 72 h postadministration.

All blood samples were transferred into heparin-treated centrifuge tubes and centrifuged for 10 min at 4,000 rpm and 4°C. All human urine samples were mixed and transferred (4 ml) into storage tubes. All biological samples were stored at −80°C.

### Bioanalytical Method Validation and Samples Determination

Separate bioanalytical methods were employed for the analysis of naringin and naringenin in rat, dog, human plasma, and human urine and faecal samples. For the rat and dog plasma samples, quercetin 3-β-D-glucoside was used as an internal standard (IS) and for the human plasma, urine, and fecal samples naringin-D4 and naringenin-D4 were used as IS's. Three concentrations served as the quality control (QC) samples in these methods: one within three folds of the lower limit of quantification (LLOQ), low QC (LQC) sample, one near the center, middle QC (MQC) sample, and one near the upper boundary of the standard curve, high QC (HQC) sample, as shown in **Tables S1** and **S2**.

In selectivity, blank samples from the appropriate biological matrices (rat plasma, dog plasma, and human plasma, urine, and feces) were obtained from at least six sources and tested for interference. The interference in each blank sample was evaluated *via* comparison with the respective LLOQ response. Furthermore, the peak area of interference should be within 20% of the analyte area and 5% that of IS at corresponding retention times in LLOQ.

Accuracy and precision were measured using six determinations per concentration at four concentrations, including the LLOQ, LQC, MQC, and HQC, which were performed in at least three separate runs for at least two days. Accuracy is the percentage difference from the added concentration and referred to as %Deviation. It is calculated as ([measured concentration/added concentration] * 100) – 100 with negative %Deviation representing underestimation and positive %Deviation representing overestimation of the true value. Precision is the variation coefficient of the replicates. It is calculated as (standard deviation of measured concentrations/mean of measured concentrations) * 100. The dilution integrity experiments were conducted using samples five times the concentration of the upper limit of quantification (ULOQ), named dilution QC. The dilution QC samples were diluted to 1/10 and 1/100 dilutions with interference-free biological matrices. The accuracy and precision of the dilution QC samples were evaluated to validate the dilution integrity. The acceptance criteria were set as follows: (i) mean values for accuracy should be ±15% of the actual QC values and ±20% of the actual LLOQ values; (ii) precision should be ≤15% for QCs and ≤20% for LLOQ.

A blank sample (matrix sample processed without IS) and a zero sample (matrix sample processed with IS) were analyzed, but not included in the calibration curve. The calibration curve consisted of at least eight samples containing naringin, naringenin, and IS covering the expected range (**Table S1**). The following acceptance criteria should be met: (i) accuracy is ±15% and precision is ≤15% of the calibration curve levels and accuracy is ±20% and precision is ≤20% for the LLOQ; (ii) at least six out of eight acceptable standards in a calibration curve. The linearity of each calibration curve was determined by plotting the peak area ratio (y) of the analyte/IS vs. nominal concentration of the analyte (x). The calibration curves were constructed by weighted (1/x^2^) least-squares linear regression.

Recovery experiments were performed by comparing the analytical results for extracted samples at three concentrations (LQC, MQC, and HQC) with unextracted standard solutions. The matrix effect experiments were performed using at least six blank matrix sources at two concentrations (LQC, HQC). The matrix effect was assessed using the ratio between the extracted blank matrix spiked with standard solution and unextracted standard solutions. The difference should be within 15% in recovery and matrix effect experiments.

The stability evaluations included the expected sample handling and storage conditions, including freeze and thaw stability (four cycles), short-term stability (room temperature for 24 h before handling; the longest batch reinjection for 72 h at 5°C, processed sample for 72 h at 5°C, and whole blood at room temperature for 1 h), long-term stability (1 and 12 months at −80°C and −20°C), and standard solution stocking stability (at least 2 weeks at −20°C). Incurred sample reanalysis was performed in separate runs on different days.

For the pharmacokinetic and excretion studies, the plasma and urine samples (rat plasma, 50 μl; dog plasma, 100 μl; human plasma, 200 μl; human urine, 200 μl) were incubated with β-glucuronidase (37°C for 2 h) to convert conjugates into naringin and naringenin. The optimal volume and activity in unit volume of enzyme solution [0.2 M acetic acid/water solution: 0.2 M ammonium acetate/water solution, 15:35 (v/v)] were determined through a pretest of the enzyme (rat plasma, 10 μl, 10 U μl^-1^; dog plasma, 10 μl, 10 U μl^-1^; human plasma, 20 μl, 20 U μl^-1^; human urine, 10 μl, 10 U μl^-1^). After enzyme incubation, 10 μl of IS solution was added to the plasma and urine samples (2 μg ml^-1^ quercetin 3-β-D-glucoside for rat and dog plasma; 583 ng ml^-1^ naringin-D4 and 160 ng ml^-1^ naringenin-D4 for human plasma; and 9,710 ng ml^-1^ naringin-D4 and 6,000 ng ml^-1^ naringenin-D4 for human urine). To each fecal sample, 9-fold methanol-normal saline (2:1, v/v) was added, mixed (18,000 rpm, 15 min) by vortexing and centrifuged (10 min, 9,000 rpm, 4°C). Next, 10 μl IS solution (971 ng ml^-1^ naringin-D4 and 600 ng ml^-1^ naringenin-D4) was added to the supernatants of feces (200 μl) without enzyme incubation. Then, all plasma, urine, and fecal samples were extracted with ethyl acetate (rat plasma, 800 μl; dog plasma, 1,000 μl; human plasma, 3,000 μl; human urine, 3,000 μl; human feces, 3,000 μl) for at least 3 min by vortexing, centrifuged for 10 min at 3,900 rpm and 4°C; supernatants were transferred into centrifuge tubes and dried with N_2_. The solid material was dissolved with acetonitrile-water (1:1, v/v) and injected into the ultra high-performance liquid chromatography coupled with triple quadrupole mass spectrometry (UHPLC/MS/MS) system as described below.

The analysis of nonclinical and clinical samples was performed with a UHPLC/MS/MS system that consisted of a Shimadzu Nexera X2 ultra high-performance liquid chromatography (UHPLC) (Shimadzu Corp., Kyoto, Japan) coupled with an AB Sciex API4000 triple-quadrupole mass spectrometry (MS/MS) system (AB Sciex, Foster City, CA, USA) equipped with an electrospray ionization source and controlled by Analyst (v. 1.6.2) software. Naringin and naringenin were separated using a Waters Xselect HSST3 C_18_ column (2.1 mm × 100 mm, 5 μm) at 40°C. The mobile phase consisted of 0.1% formic acid (v/v) in water (A) and 0.1% formic acid in acetonitrile (B) using a linear gradient of 90% A (0–0.5 min), 90 to 60% A (0.5–1.5 min), 60% to 40% A (1.5–3.5 min), 40% to 10% A (3.5–4.5 min), 10% A (4.5–5.5 min), 10% to 90% A (5.5–6.0 min), and 90% A (6.0–7.5 min) for plasma; and 80% A (0–0.2 min), 80 to 64% A (0.2–2.0 min), 64% to 63% A (2.0–4.4 min), 63% to 0% A (4.4–4.5 min), 0% A (4.5–5.5 min), 0% to 90% A (5.5–5.6 min), 90% A (5.6–5.9 min), 90% to 35% A (5.9–6.0 min), 35% A (6.0–6.2 min), 35 to 80% A (6.2–6.3 min), and 80% A (6.3–7.5 min) for urine and feces. The injection volume was 10 μl with the flow rate of 0.3 ml min^-1^ for plasma, and 0.4 ml min^-1^ for urine and feces. The compounds were detected by multiple reaction monitoring in the negative ion electrospray mode. The electrospray source conditions were as follows: ion source gas1 30 psi, ion source gas2 60 psi, curtain gas 20 psi, collisionally activated dissociation (CAD) gas 8 psi, temperature 500°C, and ion spray voltage −4,500 V. Zero air was used for ion source gas1 and ion source gas2 and nitrogen for curtain gas and CAD gas. The collision conditions for the compounds were as follows: naringin, 579.2→271.0 (*m/z*), declustering potential (DP) −110 V, entrance potential (EP) −9 V, collision energy (CE) −48 V, and collision cell exit potential (CXP) −8 V; naringenin, 271.0→150.9 (*m/z*), DP −91 V, EP −9 V, CE −25 V, and CXP −7 V; quercetin 3-β-D-glucoside, 463.0→299.9 (*m/z*), DP −100 V, EP −10 V, CE −37 V, and CXP −7 V; naringin-D4, 583.4→275.2 (*m/z*), DP −100 V, EP −8 V, CE −45 V, and CXP −8 V; and naringenin-D4, 275.0→151.0 (*m/z*), DP −90 V, EP −8 V, CE −30 V, and CXP −6 V.

All bioanalytical method validation, determination, and procedures were performed in accordance with the Technical Guidelines for Nonclinical Pharmacokinetic Studies of Drugs, Drug Clinical Trial Quality Management Standards, the Guidelines for Laboratory Management of Biological Samples Analysis in Clinical Trials, and the Technical Guidelines for Clinical Pharmacokinetic Studies of Drugs, published by the China Food and Drug Administration, the FDA (CDER) Guidance for the Industry Bioanalytical Method Validation (Rockville, MD, USA, 2001), and the Guideline on Bioanalytical Method Validation (Chinese Pharmacopoeia Commission, 2015).

### Metabolic Studies in Rat and Human Liver and Kidney Microsomes

For incubation system I-rat, naringin solution (1 mM, 10 μl), NADPH-A (50 μl), rat liver microsomes (25 μl), phosphate solution (200 μl), and deionized water (705 μl) were mixed and incubated for 5 min in a water bath shaker (37°C). Then, NADPH-B (10 μl) was added and incubated for 2 h. For incubation system II-rat, naringin solution (1 mM, 10 μl), D-gluconate-1, 4-lactone solution (50 μl), rat liver microsomes (25 μl), phosphate solution (200 μl), alamethicin (10 μl), and deionized water (655 μl) were mixed and incubated for 5 min in a water bath shaker (37°C). Then, UDPGA (50 μl) was added and incubated for 2 h. For incubation system III-rat, naringin solution (1 mM, 10 μl), D-gluconate-1, 4-lactone solution (50 μl), rat kidney microsomes (147 μl), phosphate solution (200 μl), alamethicin (10 μl), and deionized water (533 μl) were mixed and incubated for 5 min in a water bath shaker (37°C). Then, UDPGA (50 μl) was added and incubated for 2 h. In addition, the naringenin solution (1 mM, 10 μl) was evaluated in rat systems I, II, and III as above.

For incubation system I-human, naringin solution (1 mM, 10 μl), NADPH-A (50 μl), human liver microsomes (50 μl), phosphate solution (200 μl), and deionized water (680 μl) were mixed and incubated for 5 min in a water bath shaker (37°C). Then, NADPH-B (10 μl) was added and the mixed solution incubated for 2 h. For incubation system II-human, naringin solution (1 mM, 10 μl), D-gluconate-1, 4-lactone solution (50 μl), human liver microsomes (50 μl), phosphate solution (0.5 M, 200 μl), alamethicin (10 μl), and deionized water (630 μl) were mixed and incubated for 5 min in a water bath shaker (37°C). Next, UDPGA (50 μl) was added and the mixed solution incubated for 2 h. In addition, the naringenin solution (1 mM, 10 μl) was evaluated in human systems I and II as above.

The incubated solution (500 μl) of systems I-rat, II-rat, III-rat, I-human, and II-human were extracted with ethyl acetate (500 μl) for at least 3 min by vortexing and centrifuged for 10 min at 13,000 rpm and 4°C. The extraction process described above was performed twice and all supernatants were transferred into centrifuge tubes, dried with N_2_, and dissolved with methanol-water (1:1, v/v). All samples were injected into a UPLC/Q-TOF-MS system consisting of a Waters Acquity ultra performance liquid chromatography (UPLC) coupled with a Micromass micro™ quadrupole time-of-flight mass spectrometry (Q-TOF-MS) system (Waters Corp., Milford, MA, USA), equipped with an electrospray ionization source, and controlled by the MassLynx (v. 4.1) software. Separation was performed on a Waters Acquity UPLC BEH C18 column (50 mm × 2.1 mm, 1.7 μm) at 40°C. The mobile phase consisted of 0.1% formic acid (v/v) in water (A) and methanol (B) using a linear gradient of 95% to 60% A (0–25 min), 60% to 40% A (25–35 min), 40% to 10% A (35–40 min), 10% A (40–43 min), 10% to 95% A (43–44 min), and 95% A (44–45 min). The injection volume was 5 μl with flow rate of 0.2 ml min^-1^. The Q-TOF-MS worked in a full scan mode (100–1,200 *m/z*), selection ion mode, and product ion mode, in negative ion mode, respectively. The conditions were as follows: capillary voltage 3,000 V, sample cone voltage 30 V, extraction cone voltage 2.0 V, source temperature 100°C, desolvation temperature 350°C, cone gas (N_2_) flow rate of 40 L/h, desolvation gas (N_2_) flow rate of 700 L/h, low collision energy 6.0 eV, and high collision energy 20–80 eV. Compounds were detected with accurate mass using leucine enkephalin for mass correction at 554.2615 (*m/z*) in negative ionization mode.

For incubation system III-human, naringin solution (25 mM, 1.5 μl), blank solution or inhibitor working solution (1.5 μl), human liver microsomes (2.25 μl), and phosphate solution (100 mM, 264.75 μl) were mixed in a tube and incubated for 5 min in a water bath shaker (37°C). Five single inhibitor working solutions were separately added. Their concentrations were as follows: α-naphthoflavone (0.04 mM) for CYP 1A2, sulfaphenazole (0.8 mM) for CYP 2C9, (+/-)-N-3-benzylnirvanol (0.8 mM) for CYP 2C19, quinidine (0.08 mM) for CYP 2D6, and ketoconazole (0.04 mM) for CYP 3A4/5. All protein concentrations were 0.15 mg ml^-1^ in the incubation system, except 0.3 mg ml^-1^ for the sulfaphenazole working solution. Then, NADPH (10 mM, 30 μl) was added and the mixed solution incubated for 45 min. Finally, cold acetonitrile (600 μl, containing 100 ng ml^-1^ flufenamic acid) was added to every tube, mixed by vortexing, centrifuged for 30 min at 14,000 g and 4°C, and the supernatants transferred into centrifuge tubes and injected into the UHPLC/MS/MS system as described above. Incubations were run in triplicate experiments. Additionally, the naringenin solution (25 mM, 1.5 μl) was also analyzed in system III as the naringin solution (25 mM, 1.5 μl) above.

To analyze compounds in incubation system III-human, a Luna C_18_ column (Phenomenex Inc., Torrance, CA, USA; 2.0 mm × 50 mm, 5 μm) was used at 40°C. The mobile phase consisted of 0.1% formic acid (v/v) in water (A) and 0.1% formic acid in acetonitrile (B). Naringin, naringenin, and their metabolites, including rhoifolin, neoeriocitrin, apigenin, eriodictyol, and 5, 7-hydroxytryptophan, were separated using a linear gradient of 10% B (0–0.3 min), 10% to 100% B (0.3–0.5 min), and 100% B (0.5–2.8 min). The injection volume was 10 μl with flow rate of 0.2 ml min^-1^. The compounds were detected by multiple reaction monitoring in the negative ion electrospray mode. The conditions were as follows: ion source gas1, 50 psi; ion source gas2, 50 psi; curtain gas, 20 psi; CAD gas, 8 psi; temperature 400°C, ion spray voltage −4,000 V. The collision conditions for compounds were as follows: naringin 579.2→271.0 (*m/z*), DP −110 V, EP −9 V, CE −48 V, and CXP −8 V; naringenin 271.0→150.9 (*m/z*), DP −91 V, EP −9 V, CE −25 V, and CXP −7 V; rhoifolin 577.2→269.1 (*m/z*), DP −132 V, EP −10 V, CE −50 V, and CXP −7 V; neoeriocitrin 595.2→287.2 (*m/z*), DP −110 V, EP −10 V, CE −43 V, and CXP −7 V; apigenin 268.9→116.9 (*m/z*), DP −96 V, EP −10 V, CE −48 V, and CXP −7 V; eriodictyol 286.9→150.9 (*m/z*), DP −75 V, EP −10 V, CE −21 V, and CXP −7 V; and 5,7-hydroxytryptophan 176.9→133.1 (*m/z*), DP −102 V, EP −10 V, CE −27 V, and CXP −7 V.

### Data and Statistical Analysis

The results were calculated and analyzed with Microsoft Excel (Version 2016, Microsoft Corp., Redmond, WA, USA) and reported in three significant figures. All pharmacokinetic and excretion parameters were expressed as mean ± standard deviation (SD). All mean and SD values were reported to three significant figures. The pharmacokinetic parameters were calculated using the Phoenix WinNonlin software (Version 6.4, Pharsight Corp., Cary, NC, USA) and compared using the Student's *t*-test (p < 0.05) in SPSS 18.0 (SPSS Inc., Chicago, IL, USA).

## Results

### Bioanalytical Method Validation

Separate bioanalytical methods were used for the measurement of naringin and naringenin in rat and dog plasma, and human plasma, urine, and feces (**Tables S1–S4**). Naringin and naringenin were determined within the nominal ranges of 4.89–9.78×10^2^ ng ml^-1^ (rats, naringin), 2.04–1.02×10^3^ ng ml^-1^ (rats, naringenin), 2.04–1.02×10^3^ ng ml^-1^ (dogs, naringin), 2.04–1.02×10^3^ ng ml^-1^ (dogs, naringenin), 5.00×10^-1^–2.00×10^2^ ng ml^-1^ (human plasma, naringin), 2.00–2.00×10^2^ ng ml^-1^ (human plasma, naringenin), 1.00×10–2.00×10^3^ ng ml^-1^ (human urine, naringin), 1.00×10^2^–1.60×10^4^ ng ml^-1^ (human urine, naringenin), 1.00–2.00×10^2^ ng ml^-1^ (human feces, naringin), and 1.00×10–1.60×10^3^ ng ml^-1^ (human feces, naringenin). Biological samples above the upper limit of quantitation were diluted 10-fold or 100-fold and reassayed. Across studies, overall precision was high, that is, all values were ≤15% for QCs and ≤20% for LLOQ (rats, naringin, ≤13.0% and naringenin, ≤13.3%; dogs, naringin, ≤8.42% and naringenin, ≤6.94%; human plasma, naringin, ≤12.3% and naringenin, ≤7.45%; human urine, naringin, ≤8.34% and naringenin, ≤10.5%; human feces, naringin, ≤14.9% and naringenin, ≤15.6%); and %Deviation was low, i.e. all mean values were ±15% of the actual QC values and ±20% of actual LLOQ values (rats, naringin ranging between −9.46%–10.6% and naringenin −11.8%–12.7%; dogs, naringin ranging between −5.56%–6.04% and naringenin −8.88%–5.10%; human plasma, naringin ranging between −11.3%–6.60% and naringenin −12.5%–9.11%; human urine, naringin ranging between −11.3%–5.63% and naringenin −15.9%–15.0%; human feces, naringin ranging between −9.90%–10.3% and naringenin −1.60%–12.7%). All differences of matrix factor (rats, naringin, ≤8.23% and naringenin, ≤4.12%; dogs, naringin, ≤5.90% and naringenin, ≤4.52%; human plasma, naringin, ≤2.56% and naringenin, ≤1.21%; human urine, naringin,≤1.59% and naringenin, ≤3.72%; human feces, naringin, ≤1.83% and naringenin, ≤2.11%) and recovery (rats, naringin, ≤7.77% and naringenin, ≤5.82%; dogs, naringin, ≤2.81% and naringenin, ≤7.04%; human plasma, naringin, ≤10.1% and naringenin, ≤4.57%; human urine, naringin, ≤9.96% and naringenin, ≤9.84%; human feces, naringin, ≤2.30% and naringenin, ≤3.77%) did not exceed 15%.

All stability test results were within 15% of nominal concentrations, including freeze and thaw (four cycles), short-term (room temperature for 24 h before handling, the longest batch reinjection for 72 h at 5°C, processed sample for 72 h at 5°C, and whole blood at room temperature for 1 h), long-term (1 and 12 months at −80°C and −20°C), and standard stock solution (at least 2 weeks at −20°C) stabilities. The recovery, interference, matrix effect, and ruggedness were evaluated to satisfy requirements of the FDA (CDER) Guidance for the Industry Bioanalytical Method Validation (Rockville, MD, USA, 2001) and Guideline on Bioanalytical Method Validation (Chinese Pharmacopoeia Commission, 2015). Incurred sample reanalyses were performed, according to pertinent guidelines; more than two-thirds of the incurred sample reanalyses results were within 20%.

### Nonclinical and Clinical Pharmacokinetics and Species Differences

**Figure 1:**
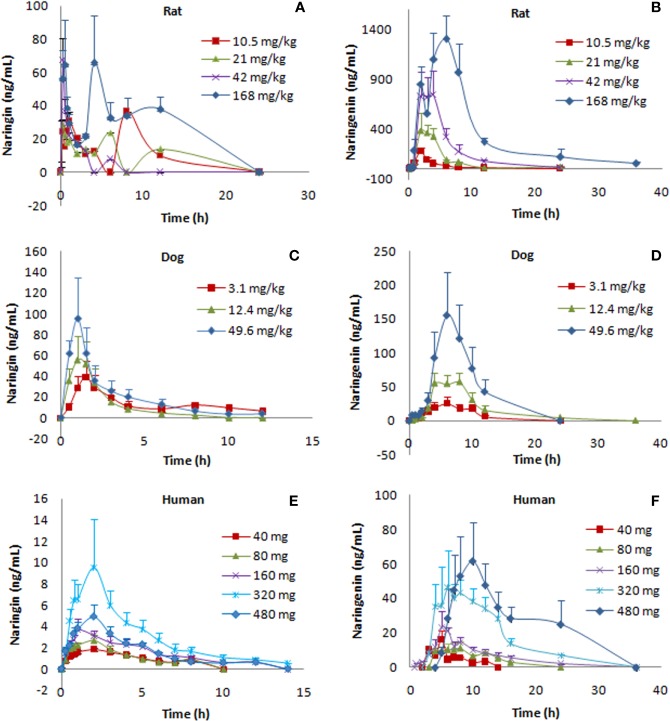
Plasma level-time curves for naringin and naringenin after oral administration (p.o.) of naringin at different doses in rats, dogs, and humans (*n*=10 per group of rats, *n*=6 per group of dogs, *n*=8 per group of humans, mean ± SE). The PK profiles were presented as follows: naringin **(A)** and naringenin **(B)** PK profiles of naringin (10.5, 21, 42, and 168 mg kg-1) in rats; naringin **(C)** and naringenin **(D)** PK profiles of naringin (3.1, 12.4, and 49.6 mg kg-1) in dogs; naringin **(E)** and naringenin **(F)** PK profiles of naringin (40, 80, 160, 320, and 480 mg) in humans.

For the single-dose studies, noncompartmental pharmacokinetic analyses were performed to obtain the following data: the area under the curve to the last quantifiable concentration (AUC_0-t_) and to infinity (AUC_0-∞_), the time for peak plasma level (*T_max_*), the peak plasma level (*C*_max_), the terminal elimination half-life (*t*_1/2_), the apparent volume of distribution (Vd), the apparent clearance (CL), and the mean residence time (MRT) (**Tables 1** and **2**). For the multiple-dose studies, pharmacokinetic parameters included the mean plasma level at steady state, *T*_max_, *t_1/2_*, the percent fluctuation, and the accumulation index (**Table 3**). **Figure 1** shows the plasma concentration-time profiles of naringin and naringenin in rats, dogs, and humans. In addition, we compared the pharmacokinetic parameters obtained from males and females (**Tables 4**–**6**).

**Table 1:** Pharmacokinetic parameters of naringin in single-dose studies (*n*=10 per groups of rats, *n*=6 per groups of dogs, *n*=8 per groups of humans, mean ± SD).

Species	Dose ^a^ (mg kg^-1^ or mg)	The route of adminstration	*t_1/2_* (h)	*T_max_* (h)	*C_max_* ^b^ (ng ml^-1^ or μg ml^-1^)	AUC_0-_*_t_* ^c^ (h·ng ml^-1^ or h μg ml^-1^)	AUC_0-∞_^c^ (h·ng ml^-1^ or h μg ml^-1^)	Vd (L/kg)	CL (L/h/kg)	MRT_0-t_ (h)	MRT_0-∞_ (h)
Rat											
	10.5	p.o.	0.328 ± 0.275	1.50 ± 2.35	31.7 ± 13.2	32.7 ± 27.7	35.7 ± 29.3	174 ± 98.3	624 ± 589	0.741 ± 0.423	1.22 ± 0.297
	21	p.o.	0.500 ± 0.493	1.53 ± 2.36	30.5 ± 16.3	34.2 ± 20.3	38.4 ± 21.4	322 ± 239	759 ± 753	1.18 ± 0.399	1.42 ± 0.621
	42	p.o.	0.463 ± 0.598	1.18 ± 1.78	52.6 ± 78.8	40.7 ± 51.3	45.1 ± 55.4	586 ± 394	1,410 ± 1,110	1.10 ± 0.381	1.24 ± 0.292
	168	p.o.	0.738 ± 0.781	2.85 ± 4.06	104 ± 77.5	140 ± 66.3	145 ± 66.4	639 ± 657	810 ± 417	2.01 ± 0.451	1.96 ± 1.08
	42	i.v.	1.62 ± 1.18	0.030 ± 0.000	88.5 ± 51.0	18.1 ± 7.58	18.1 ± 7.55	8.29 ± 10.0	2.90 ± 1.66	0.496 ± 0.382	0.570 ± 0.476
Dog											
	3.1	p.o.	1.84 ± 1.46	1.33 ± 0.258	39.9 ± 10.4	108 ± 58.8	129 ± 78.2	61.2 ± 23.0	31.2 ± 15.4	2.50 ± 1.05	3.38 ± 1.88
	12.4	p.o.	1.31 ± 0.581	1.00 ± 0.316	70.2 ± 16.9	124 ± 32.2	135 ± 35.5	171 ± 59.4	98.0 ± 27.2	1.77 ± 0.371	2.17 ± 0.633
	49.6	p.o.	1.31 ± 0.293	1.00 ± 0.447	107 ± 78.2	211 ± 92.1	224 ± 104	482 ± 173	267 ± 127	2.46 ± 1.11	2.73 ± 1.02
	12.4	i.v.	1.37 ± 0.347	0.080 ± 0.000	5.80 ± 0.946	2.92 ± 0.406	2.93 ± 0.403	8.69 ± 3.35	4.31 ± 0.604	0.703 ± 0.153	0.727 ± 0.143
Human											
	40	p.o.	2.48 ± 1.64	1.97 ± 1.11	2.24 ± 0.670	7.61 ± 3.50	9.99 ± 4.22	275 ± 167	67.0 ± 36.3	2.66 ± 0.443	4.90 ± 1.69
	80	p.o.	1.90 ± 1.22	2.09 ± 1.39	2.94 ± 1.17	9.27 ± 3.37	12.2 ± 3.19	295 ± 117	103 ± 49.9	2.80 ± 0.543	3.83 ± 1.69
	160	p.o.	3.64 ± 3.01	2.50 ± 1.41	4.29 ± 2.63	15.7 ± 7.51	22.4 ± 9.36	725 ± 568	144 ± 61.6	3.30 ± 1.12	6.54 ± 4.41
	160 (high- fat diet) ^d^	p.o.	3.11 ± 1.95	2.66 ± 1.87	3.04 ± 1.15	12.8 ± 4.74	18.2 ± 5.81	755 ± 509	172 ± 64.3	3.45 ± 1.22	6.19 ± 2.77
	320	p.o.	2.51 ± 1.58	1.66 ± 0.990	10.9 ± 12.4	37.5 ± 32.2	39.8 ± 32.2	677 ± 505	202 ± 131	3.17 ± 0.677	4.01 ± 1.05
	480	p.o.	2.01 ± 0.498	1.66 ± 0.834	5.69 ± 2.80	19.4 ± 7.86	21.2 ± 7.95	1,360 ± 992	477 ± 372	3.08 ± 0.704	3.86 ± 0.779

a The unit was mg kg^-1^ for rat and dog, and mg for human.

b The unit was μg ml^-1^ for i.v. administration groups, and ng ml^-1^ for p.o. administration groups.

c The unit was h μg ml^-1^ for i.v. administration groups, and h ng ml^-1^ for p.o. administration groups.

d Compared to 160 mg group, the acceptable level of significance was established at P < 0.05*.

**Table 2:** Pharmacokinetic parameters of naringenin in single-dose studies (*n*=10 per groups of rats, *n*=6 per groups of dogs, *n*=8 per groups of humans, mean ± SD).

Species	Dose (mg kg^-1^ or mg ^a^)	The route of adminstration	*t_1/2_* (h)	*T_max_* (h)	*C_max_^b^* (ng ml-1 or μg ml-1)	AUC_0-t_ ^c^ (h·ng ml-1 or h μg ml-1)	AUC_0-∞_^c^ (h·ng ml-1 or h μg ml-1)	Vd (L/kg)	CL (L/h/kg)	MRT_0-t_ (h)	MRT_0-∞_ (h)
Rat											
	10.5	p.o.	2.08 ± 1.30	2.50 ± 1.27	0.187 ± 0.114	0.518 ± 0.207	0.541 ± 0.216	74.2 ± 64.4	25.1 ± 18.1	3.84 ± 1.61	4.30 ± 1.97
	21	p.o.	1.93 ± 1.14	3.00 ± 1.41	0.539 ± 0.487	1.68 ± 1.14	1.71 ± 1.15	57.5 ± 57.0	18.4 ± 12.5	5.02 ± 2.90	5.22 ± 3.02
	42	p.o.	2.47 ± 1.33	3.30 ± 1.34	1.08 ± 0.745	4.16 ± 3.80	4.22 ± 3.78	66.6 ± 67.9	18.7 ± 16.6	5.20 ± 1.45	5.55 ± 1.64
	168	p.o.	2.75 ± 1.04	5.70 ± 2.31	1.83 ± 0.858	11.3 ± 4.24	11.5 ± 4.26	93.4 ± 121	19.6 ± 17.3	7.73 ± 2.25	8.03 ± 2.25
	42	i.v.	2.45 ± 1.38	0.040 ± 0.021	3.98 ± 2.51	2.36 ± 1.12	2.39 ± 1.11	88.3 ± 113	23.4 ± 16.0	3.36 ± 2.11	3.66 ± 2.14
Dog											
	3.1	p.o.	2.42 ± 1.15	5.67 ± 0.816	0.033 ± 0.013	0.166 ± 0.063	0.191 ± 0.064	70.0 ± 63.8	18.0 ± 6.73	6.33 ± 1.41	7.67 ± 2.20
	12.4	p.o.	1.93 ± 0.890	6.00 ± 1.79	0.082 ± 0.025	0.408 ± 0.160	0.463 ± 0.197	81.4 ± 32.8	32.2 ± 16.4	6.80 ± 1.53	7.57 ± 2.30
	49.6	p.o.	1.74 ± 0.547	6.33 ± 2.34	0.204 ± 0.053	1.11 ± 0.697	1.15 ± 0.678	132 ± 74.2	51.7 ± 18.8	6.97 ± 1.63	7.37 ± 1.65
	12.4	i.v.	1.62 ± 1.32	5.25 ± 3.57	34.8 ± 13.6	0.142 ± 0.094	0.158 ± 0.101	224 ± 124	178 ± 202	4.35 ± 2.56	5.14 ± 3.34
Human											
	40	p.o.	1.26 ± 1.06	6.86 ± 2.54	11.7 ± 12.4	21.6 ± 22.9	23.9 ± 22.6	61.5 ± 38.4	41.5 ± 33.1	5.25 ± 0.583	6.30 ± 4.77
	80	p.o.	3.17 ± 4.24	8.38 ± 4.24	12.1 ± 8.52	45.2 ± 37.2	72.6 ± 87.8	174 ± 158	75.9 ± 103	8.01 ± 4.88	14.3 ± 23.3
	160	p.o.	1.50 ± 0.838	9.13 ± 2.59	26.2 ± 18.2	91.0 ± 70.6	97.8 ± 71.5	60.6 ± 22.1	31.2 ± 16.1	9.82 ± 4.20	10.1 ± 5.05
	160 (high- fat diet) ^d^	p.o.	2.63 ± 1.30	8.88 ± 3.36	23.3 ± 17.7	93.6 ± 52.1	164 ± 97.4	78.4 ± 20.6	22.9 ± 5.99	9.54 ± 2.64	9.56 ± 2.93
	320	p.o.	2.67 ± 1.54	9.00 ± 2.39	73.6 ± 45.6	409 ± 118	452 ± 110	47.0 ± 30.6	6.00 ± 2.23	10.2 ± 2.47	11.1 ± 3.27
	480	p.o.	3.37 ± 3.38	13.1 ± 5.17	96.8 ± 76.5	615 ± 348	805 ± 463	55.8 ± 47.4	6.32 ± 7.94	12.0 ± 2.42	12.5 ± 3.34

a The unit was mg kg^-1^ for rats and dogs, and mg for humans.

b The unit was μg ml^-1^ for rats and dogs, and ng ml^-1^ for humans.

c The unit was h μg ml^-1^ for rat and dogs, and h ng ml^-1^ for humans.

d Compared to 160 mg group, the acceptable level of significance was established at P < 0.05*.

**Table 3:** Pharmacokinetic parameters of naringin and naringenin in multiple-dose studies (p.o., three times a day, *n*=10 per groups of rats, *n*=6 per groups of dogs, *n*=8 per groups of humans, mean ± SD).

Species	Dose (mg kg^-1^ or mg ^a^)	Analyte	*t_1/2_* (h)	*T_max_* (h)	$C_{av}^{SS}$ g ml^-1^)	*Fluctuation* ^b^ (%)	*Accumulation* ^c^ *Index*
Rat							
	42	Naringin	0.320 ± 0.191	1.70 ± 1.47	11.7 ± 15.9	728 ± 711	1.22 ± 0.401
	42	Naringenin	2.81 ± 1.84	3.90 ± 2.28	389 ± 415	184 ± 114	1.31 ± 0.625
Dog							
	12.4	Naringin	0.938 ± 0.353	1.67 ± 0.753	18.6 ± 4.68	306 ± 103	1.21 ± 0.099
	12.4	Naringenin	2.17 ± 1.03	6.00 ± 0.000	49.8 ± 12.2	72.7 ± 57.2	1.35 ± 0.209
Human							
	160	Naringin	2.94 ± 2.01	1.66 ± 1.62	1.75 ± 0.365	187 ± 70.7	1.22 ± 0.285
	160	Naringenin	3.14 ± 2.01	5.83 ± 1.17	39.0 ± 38.7	177 ± 76.5	1.21 ± 0.277

a The unit was mg kg^-1^ for rats and dogs, and mg for humans.

b $Fluctuation=\frac{\left(C_{max}^{SS}-C_{min}^{SS}\right)}{C_{av}^{SS}}\times 100\%$.

c $Accumulation\;Index=\frac{{\text{AUC}}_{0-24}^{SS}}{{\text{AUC}}_{0-24}^{Day\;1}}$

**Table 4:** The pharmacokinetic parameters of naringin for different sexes in single-dose studies (half male and female for rats and dogs, a mix of males and females for humans, mean ± SD).

Information of administration ^a^		Male					Female ^b^				
		*t_1/2_* (h)	*T_max_* (h)	*C_max_* ^c^ (ng ml^-1^ or μg ml^-1^)	AUC_0-_*_t_* ^d^ (h·ng ml^-1^ or h μg ml^-1^)	AUC_0-∞_^d^ (h·ng ml^-1^ or h μg ml^-1^)	*t_1/2_* (h)	*T_max_* (h)	*C_max_* ^c^ (ng ml^-1^ or μg ml^-1^)	AUC_0-_*_t_* ^d^ (h·ng ml^-1^ or h μg ml^-1^)	AUC_0-∞_^d^ (h·ng ml^-1^ or h μg ml^-1^)
***Rat***											
10.5	p.o.	0.190 ± 0.063	1.95 ± 3.40	36.1 ± 14.9	33.5 ± 28.8	36.9 ± 30.2	0.465 ± 0.344	1.05 ± 0.622	27.2 ± 11.2	31.9 ± 29.9	34.6 ± 31.9
21	p.o.	0.325 ± 0.381	1.50 ± 2.52	29.9 ± 18.5	21.4 ± 16.5	24.8 ± 18.0	0.675 ± 0.570	1.55 ± 2.49	31.1 ± 16.0	47.0 ± 15.6*	51.9 ± 15.7*
42	p.o.	0.375 ± 0.354	0.650 ± 0.762	92.7 ± 99.4	68.1 ± 62.7	74.9 ± 67.6	0.550 ± 0.813	1.70 ± 2.42	12.6 ± 9.42	13.3 ± 10.3	15.3 ± 11.6
168	p.o.	0.625 ± 0.781	3.75 ± 4.85	113 ± 102	144 ± 80.3	149 ± 84.3	0.850 ± 0.854	1.95 ± 3.39	95.8 ± 54.4	137 ± 50.7	142 ± 52.4
42	i.v.	1.65 ± 1.48	0.030 ± 0.000	106 ± 37.6	21.3 ± 7.03	21.4 ± 6.93	1.60 ± 0.972	0.030 ± 0.000	70.2 ± 60.0	14.8 ± 7.29	14.8 ± 7.28
***Dog***											
3.1	p.o.	2.41 ± 1.98	1.50 ± 0.231	42.9 ± 9.19	128 ± 76.6	149 ± 97.4	1.26 ± 0.633	1.17 ± 0.289	36.8 ± 12.6	88.5 ± 40.0	109 ± 67.7
12.4	p.o.	1.20 ± 0.443	1.00 ± 0.500	80.2 ± 17.2	132 ± 40.0	137 ± 42.3	1.42 ± 0.782	1.00 ± 0.100	60.2 ± 10.8	115 ± 27.9	132 ± 36.8
49.6	p.o.	1.50 ± 0.117	0.833 ± 0.577	56.6 ± 6.83	181 ± 46.7	185 ± 45.5	1.13 ± 0.312	1.17 ± 0.289	157 ± 87.5	240 ± 128	262 ± 144
12.4	i.v.	1.48 ± 0.404	0.080 ± 0.000	5.74 ± 1.05	2.64 ± 0.256	2.64 ± 0.255	1.25 ± 0.317	0.080 ± 0.000	5.86 ± 1.07	3.19 ± 0.344	3.20 ± 0.337
***Human***^**e**^											
40	p.o.	3.07 ± 1.78	1.55 ± 0.622	2.43 ± 0.727	9.39 ± 3.23	12.5 ± 3.18	1.49 ± 0.849	2.67 ± 1.53	1.92 ± 0.516	4.64 ± 1.01	5.83 ± 0.737*
80	p.o.	2.46 ± 1.83	2.75 ± 1.71	2.58 ± 1.43	8.35 ± 4.36	13.2 ± 4.16	1.48 ± 0.448	1.44 ± 0.657	3.30 ± 0.916	10.2 ± 2.28	11.5 ± 2.69
160	p.o.	2.41 ± 1.79	1.75 ± 0.500	5.14 ± 3.60	13.7 ± 9.73	17.7 ± 9.93	4.57 ± 3.65	3.25 ± 1.71	3.44 ± 1.08	17.8 ± 5.04	25.9 ± 8.47
160 ^f^	p.o.	3.86 ± 2.02	2.44 ± 1.98	2.30 ± 0.201	9.56 ± 3.57	14.7 ± 5.83	2.54 ± 1.96	2.88 ± 2.02	3.78 ± 1.26	16.1 ± 3.27*	20.9 ± 4.78

a The dose unit was mg kg^-1^ for rats and dogs, and mg for humans.

b Compared to males, the acceptable level of significance was established at P < 0.05*.

c The unit was μg ml^-1^ for i.v. administration groups, and ng ml^-1^ for p.o. administration groups.

d The unit was h μg ml^-1^ for i.v. administration groups, and h ng ml^-1^ for p.o. administration groups.

e There were four females in 40 mg group (one female, p.o., placebo), four females in 80 mg group (zero female, p.o., placebo), five females in 160 mg group (one female, p.o., placebo), five females in 160 mg (high-fat diet) group (one female, p.o., placebo), three females in 320 mg group (two females, p.o., placebo), and two females in 480 mg group (two females, p.o., placebo).

f The high-fat diet group.

**Table 5:** The pharmacokinetic parameters of naringenin for different sexes in single-dose studies (half male and female for rats and dogs, a mix of males and females for humans, mean ± SD).

Information of administration ^a^		Male					Female ^b^				
		*t_1/2_* (h)	*T_max_* (h)	*C_max_* ^c^ (ng ml^-1^ or μg ml^-1^)	AUC_0-_*_t_* ^d^ (h·ng ml^-1^ or h μg ml^-1^)	AUC_0-∞_^d^ (h·ng ml^-1^ or h μg ml^-1^)	*t_1/2_* (h)	*T_max_* (h)	*C_max_* ^c^ (ng ml^-1^ or μg ml^-1^)	AUC_0-_*_t_* ^d^ (h·ng ml^-1^ or h μg ml^-1^)	AUC_0-∞_^d^ (h·ng ml^-1^ or h μg ml^-1^)
***Rat***											
10.5	p.o.	1.33 ± 0.453	3.00 ± 1.73	0.202 ± 0.079	0.593 ± 0.161	0.617 ± 0.180	2.83 ± 1.48	2.00 ± 0.810	0.172 ± 0.151	0.443 ± 0.237	0.465 ± 0.242
21	p.o.	1.26 ± 0.327	2.00 ± 0.707	0.804 ± 0.550	2.13 ± 1.39	2.17 ± 1.41	2.60 ± 1.32	4.00 ± 1.22	0.274 ± 0.236	1.24 ± 0.694	1.26 ± 0.699
42	p.o.	1.46 ± 0.777	4.00 ± 1.41	0.818 ± 0.615	2.66 ± 1.53	2.75 ± 1.49	3.47 ± 0.958*	2.60 ± 0.894	1.34 ± 0.838	5.66 ± 4.96	5.70 ± 4.95
168	p.o.	2.77 ± 1.24	3.80 ± 1.48	1.89 ± 0.805	10.4 ± 2.58	10.6 ± 2.42	2.73 ± 0.951	7.60 ± 0.894*	1.77 ± 1.00	12.2 ± 5.64	12.4 ± 5.73
42	i.v.	1.99 ± 0.529	0.030 ± 0.000	5.54 ± 2.40	2.99 ± 0.986	3.01 ± 0.982	2.91 ± 1.87	0.043 ± 0.025	2.43 ± 1.55*	1.72 ± 0.915	1.78 ± 0.945
***Dog***											
3.1	p.o.	2.54 ± 0.931	6.00 ± 0.482	32.8 ± 5.33	195 ± 63.8	229 ± 63.3	2.29 ± 1.54	5.33 ± 1.15	32.7 ± 20.4	137 ± 58.2	152 ± 44.3
12.4	p.o.	2.30 ± 1.20	6.00 ± 2.00	68.3 ± 22.9	309 ± 128	398 ± 257	1.56 ± 0.345	6.00 ± 2.00	96.0 ± 21.8	508 ± 135	528 ± 136
49.6	p.o.	1.99 ± 0.712	6.67 ± 3.06	238 ± 44.7	1,420 ± 960	1,460 ± 914	1.50 ± 0.231	6.00 ± 2.00	169 ± 38.4	809 ± 131	842 ± 152
12.4	i.v.	1.98 ± 1.88	5.58 ± 4.19	32.1 ± 18.9	138 ± 114	163 ± 124	1.27 ± 0.677	4.92 ± 3.74	37.4 ± 9.27	145 ± 95.6	153 ± 101
***Human***^**e**^											
40	p.o.	1.13 ± 0.629	7.75 ± 3.10	4.34 ± 1.44	6.81 ± 3.46	9.34 ± 4.01	1.45 ± 1.65	5.67 ± 1.15	21.4 ± 14.4*	41.2 ± 23.1*	43.3 ± 22.6*
80	p.o.	1.98 ± 1.03	9.25 ± 4.43	8.70 ± 3.87	38.0 ± 24.2	44.4 ± 29.2	4.37 ± 6.09	7.50 ± 4.51	15.6 ± 11.1	52.4 ± 50.0	101 ± 122
160	p.o.	1.50 ± 1.14	8.25 ± 3.30	32.8 ± 24.9	110 ± 98.1	114 ± 101	1.50 ± 0.577	10.0 ± 1.63	19.5 ± 5.64	71.8 ± 32.3	81.2 ± 31.3
160^f^	p.o.	3.47 ± 1.34	7.75 ± 3.10	29.4 ± 24.2	92.9 ± 57.5	229 ± 138	2.07 ± 1.15	10.0 ± 3.65	17.2 ± 6.97	94.3 ± 55.0	121 ± 47.3

a The dose unit was mg kg^-1^ for rats and dogs, and mg for humans.

b Compared to males, the acceptable level of significance was established at P < 0.05*.

c The unit was μg ml^-1^ for rats, and ng ml^-1^ for dogs and humans.

d The unit was h μg ml^-1^ for rat, and h ng ml^-1^ for dogs and humans.

e There were four females in 40 mg group (one female, p.o., placebo), four females in 80 mg group (zero female, p.o., placebo), five females in 160 mg group (one female, p.o., placebo), five females in 160 mg (high-fat diet) group (one female, p.o., placebo), three females in 320 mg group (two females, p.o., placebo), and two females in 480 mg group (two females, p.o., placebo).

f The high-fat diet group.

**Table 6:** The pharmacokinetic parameters of naringin and naringenin for different sexes in multiple -dose studies (half male and female for rats and dogs, a mix of males and females for humans, mean ± SD).

Dose and analyte ^a^		Male					Female ^b^				
		*t_1/2_* (h)	*T_max_* (h)	$C_{av}^{SS}$. g ml^-1^)	*Fluctuation* ^c^ (%)	*Accumulation* ^d^ *Index*	*t_1/2_* (h)	*T_max_* (h)	$C_{av}^{SS}$. g ml^-1^)	*Fluctuation* ^c^ (%)	*Accumulation* ^d^ *Index*
***Rat***											
42	Naringin	0.275 ± 0.205	0.700 ± 0.411	6.03 ± 9.48	844 ± 1,030	1.08 ± 0.224	0.365 ± 0.187	2.70 ± 1.48*	17.4 ± 19.9	612 ± 233	1.38 ± 0.544
42	Naringenin	2.18 ± 0.566	3.40 ± 0.548	178 ± 90.8	104 ± 58.9	0.817 ± 0.267	3.45 ± 2.50	4.40 ± 3.29	600 ± 517	264 ± 98.2	1.79 ± 0.457*
***Dog***											
12.4	Naringin	1.13 ± 0.041	2.17 ± 0.764	15.1 ± 2.53	242 ± 8.10	1.20 ± 0.127	0.743 ± 0.443	1.17 ± 0.289	22.1 ± 3.41	370 ± 119	1.21 ± 0.092
12.4	Naringenin	1.38 ± 0.289	6.00 ± 0.000	49.0 ± 10.4	66.7 ± 82.5	1.36 ± 0.241	2.95 ± 0.839	6.00 ± 0.000	50.7 ± 16.1	78.6 ± 35.8	1.32 ± 0.224
***Human*** ^**e**^											
160	Naringin	2.67 ± 1.40	1.55 ± 1.94	1.75 ± 0.403	210 ± 79.6	1.17 ± 0.167	3.40 ± 3.12	1.83 ± 1.26	1.74 ± 0.377	149 ± 37.5	1.30 ± 0.462
160	Naringenin	3.50 ± 2.24	6.25 ± 1.26	47.2 ± 47.0	167 ± 95.7	1.29 ± 0.326	2.42 ± 1.90	5.00 ± 0.000	22.6 ± 8.42	198 ± 21.3	1.16 ± 0.205

a The dose unit was mg kg^-1^ for rats and dogs, and mg for humans.

b Compared to males, the acceptable level of significance was established at P < 0.05*.

c $Fluctuation=\frac{\left(C_{max}^{SS}-C_{min}^{SS}\right)}{C_{av}^{SS}}\times 100\%$.

d $Accumulation\;Index=\frac{{\text{AUC}}_{0-24}^{SS}}{{\text{AUC}}_{0-24}^{Day\;1}}$.

e There were four females in human multiple-dose study (one female, p.o., placebo).

Species differences were presented for the pharmacokinetic parameters. In the nonclinical p.o. administration studies, the rat and dog plasma concentrations of naringin increased rapidly to peaks within about 1 h (rats, *T*_max_, 1.77 ± 2.64 h and dogs, *T*_max_, 1.11 ± 0.344 h). The *t_1/2_* of naringin in rats was shorter than that observed in dogs (1.41 ± 0.671 h); the plasma concentrations for rats declined below 20% of *C_max_* within 1 h (naringin, *t_1/2_*, < 1 h), demonstrating a multiple-peak pattern. In clinical studies, plasma naringin concentration increased to maximum at about 2 h (*T*_max_, 2.09 ± 1.15 h) and decreased to 50% of *C_max_* at about 3 h (*t* ***_1/2_***, 2.69 ± 1.77 h). Subsequently, owing to naringin metabolism in the human intestine, naringenin showed a considerable lag-time in human plasma (5.74 ± 2.38 h) compared to observations in rats (0.701 ± 0.523 h) and dogs (1.83 ± 0.923 h); the naringenin concentrations increased to *C_max_* at about 3.62 ± 3.19 h later in humans, compared to those in rats (2.93 ± 2.01 h) and dogs (4.17 ± 1.68 h). Naringenin *t_1/2_* in humans (2.60 ± 1.89 h) differed insignificantly from values obtained in rats (2.31 ± 1.21 h) and dogs (2.03 ± 0.968 h). Although the clinical naringin administration doses concurred with animal doses, according to weight and surface, AUC and *C*_max_ in humans were significantly different from those observed in animals. In nonclinical and clinical studies, all naringin AUC values fluctuated and slightly increased, whereas those for naringenin were linear in terms of doses. The absolute bioavailability of naringin was 44.1% for rats (42 mg kg^-1^) and 34.4% for dogs (12.4 mg kg^-1^) in the forms of naringin and naringenin. In food-effect trials, the pharmacokinetic parameters of naringin and naringenin, including *t_1/2_*, *T_max_*, *C_max_*, and AUC, were insignificantly altered by a high-fat diet. As presented in **Table 3**, no significant accumulation of naringin and naringenin was observed in multiple-dose studies in rats, dogs, and humans. Furthermore, there were significant differences between the pharmacokinetic parameters of males and females in rats and humans. In single-dose studies, some pharmacokinetic parameters of females were significantly higher than those of males as follows: AUC (naringin, rats, p.o., 21 mg kg^-1^; naringin, humans, 160 mg (high-fat diet); and naringenin, humans, 40 mg), *t*_1/2_ (naringenin, rats, p.o., 42 mg kg^-1^), *T*_max_ (naringenin, rats, p.o., 168 mg kg^-1^), and *C*_max_ (naringenin, humans, 40 mg), while few pharmacokinetic parameters of females were significantly lower than those of males (AUC of naringin, humans, 40 mg; *C*_max_ of naringenin, rats, i.v., 42 mg kg^-1^). In multiple-dose studies, significantly higher parameters of females were only observed in rats (naringin, *T*_max_, 2.70 ± 1.48 h and naringenin, accumulation index, 1.79 ± 0.457).

### Metabolism in Liver and Kidney Microsomes of Rats and Humans

**Figure 2:**
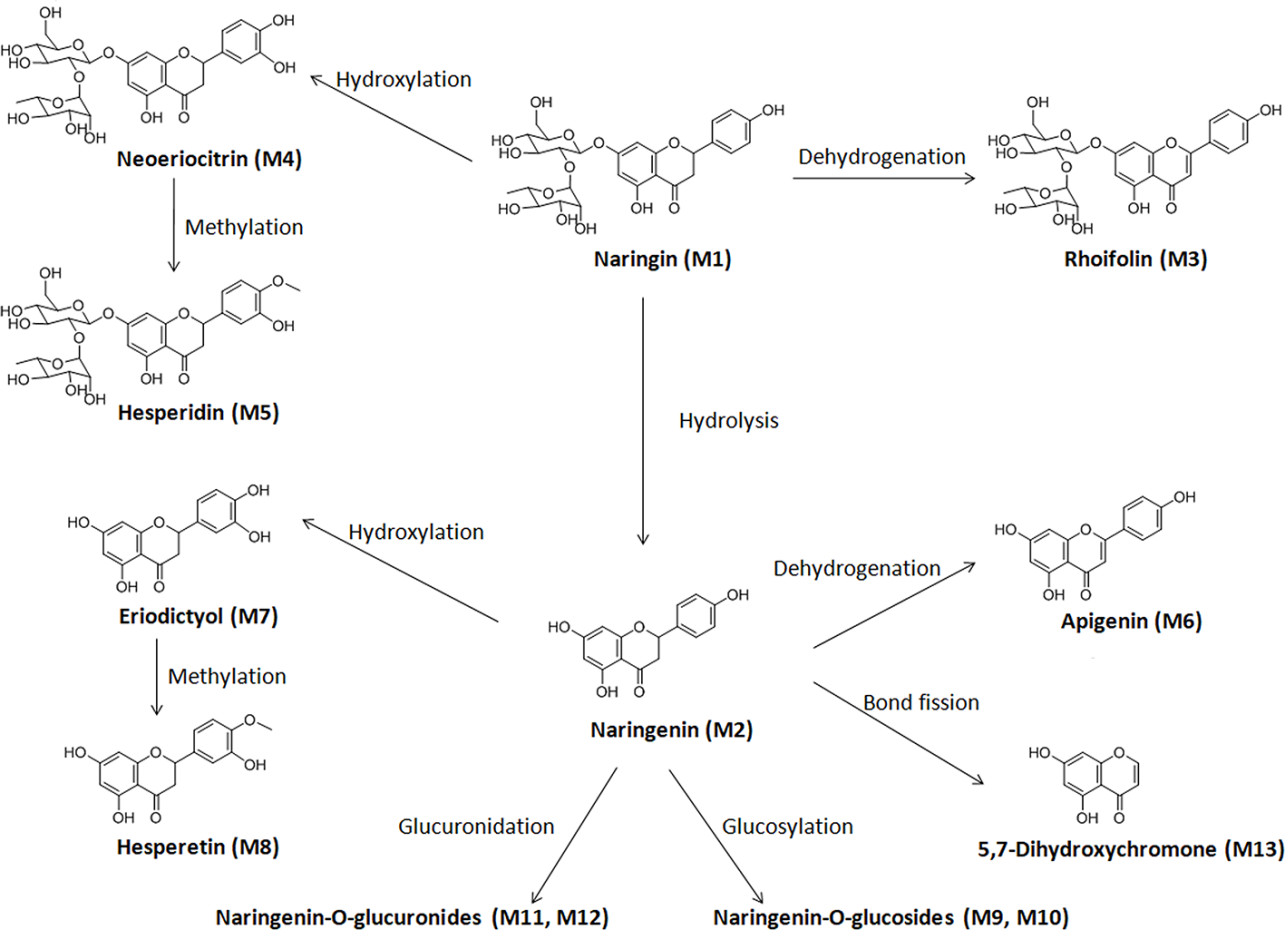
Proposed metabolic pathway of naringin in liver and kidney microsomes of rats and humans.

**Figure 3:**
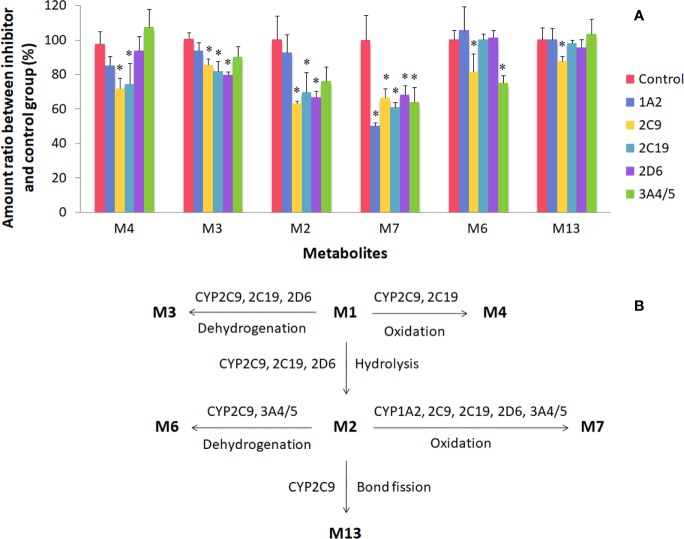
Metabolism of naringin and naringenin in human liver microsomes (incubation system III-human). There were six metabolites on the abscissa, including neoeriocitrin (M4), rhoifolin (M3), naringenin (M2), eriodictyol (M7), apigenin (M6), and 5, 7-dihydroxychromone (M13). For plot **(A)**, the control and inhibitor groups were presented as follows: blank solution, control; α-naphthoflavone groups, 1A2; sulfaphenazole, 2C9; benzylnirvanol, 2C19; quinidine, 2D6; ketoconazole, 3A4/5. *Compared to the control group, the acceptable level of significance was established at P < 0.05. For plot **(B)**, CYP enzyme pathways of naringin and naringenin were proposed in human liver microsomes.

This study focused on the phase I and II metabolism of naringin and naringenin in rat and human liver microsomes. Additionally, the metabolites were analyzed in rat kidney microsomes. Twelve metabolites of naringin and naringenin were identified in rat and human liver and kidney microsomes (**Figures S1** and **S2** and **Tables 7** and **S5**). With the exception of the methylated metabolites of naringin and naringenin, there were similar metabolites found after incubation with rat and human liver microsomes. The metabolic pathways of naringin and naringenin obtained with human liver microsomes are presented in **Figure 2**. Furthermore, six metabolites, including neoeriocitrin, rhoifolin, naringenin, eriodictyol, apigenin, and 5, 7-dihydroxychromone, were simultaneously measured after the blank solution or five single inhibitor working solutions were added to the incubation system III-human. Compared to the control groups, the number of metabolites decreased significantly to different degrees due to the addition of a single enzyme inhibitor to inhibitor groups (**Figure 3**). The results demonstrated that CYP2C9 was a major metabolic enzyme of naringin and responsible for the six metabolites. The enzyme contributions were shown sequentially as follows: CYP2C19, four metabolites; CYP2D6, three metabolites; CYP3A4/5, two metabolites; and CYP1A2, one metabolite.

**Table 7:** Metabolites of naringin (G) and naringein (S) in liver and kidney microsomes of rats and humans.

No.	Metabolism	Name	Retention time (min)	[M-H]^-^ (Error, ppm)	Main fragment ions (*m/z*)	Identified by standard	Rat microsomes			Human liver microsomes	
							Liver		Kidney		
							G	S	G/S	G	S
M1	[M_1_]	Naringin	19.19	579.1699 (−2.6)	459, 271, 151	√	+		+	+	
M2	[M_2_]	Naringenin	24.52	271.0581 (−9.2)	177, 151, 119	√	+	+	+	+	+
M3	[M_1_-2H]	Rhoifolin	21.15	577.1561 (0.7)	475, 273, 167	√	+			+	
M4	[M_1_+O]	Neoeriocitrin	16.62	595.1653 (−1.7)	459, 287, 151	√	+			+	
M5	[M_1_+O+CH_3_]	Hesperidin	20.50	609.1791 (−4.6)	459, 271, 151	√	+				
M6	[M_2_-2H]	Apigenin	28.16	269.0468 (6.7)	225, 151, 117	√		+			+
M7	[M_2_+O]	Eriodictyol	20.87	287.0561 (1.4)	151, 135, 107	√		+			+
M8	[M_2_+O+CH_3_]	Hesperetin	25.09	301.0736 (8.0)	271, 177, 151, 119	√		+			
M9	[M_2_+Glc]	Naringenin-O-glucoside	16.72	433.1175 (9.2)	271, 165, 151			+			
M10	[M_2_+Glc]	Naringenin-O-glucoside	17.67	433.1157 (5.1)	271, 165, 151			+			+
M11	[M_2_+GlcuA]	Naringenin-O-glucuronide	13.54	447.0897 (−6.7)	271, 151,			+			+
M12	[M_2_+GlcuA]	Naringenin-O-glucuronide	14.05	447.0891 (−5.8)	271, 151			+	+		
M13	Bond fission of M_2_	5,7-Dihydroxychromone	12.60	177.0168 (−5.6)	133, 109	√		+			+

### Excretion in Humans

For a mass balance study, urine and feces were collected after p.o. administration of naringin in clinical studies. The cumulative excretion percentages of naringin in humans were less than 20% in the form of naringin and naringenin (**Table 8**). To evaluate enzyme influence on the fecal naringin content and account for the entire dose administered to subjects, six fecal samples from individuals who did not receive orally administered naringin were mixed. Next, 9-fold normal saline, instead of ninefold methanol-normal saline (2:1, v/v), was added to maintain enzyme activity in the feces. Subsequently, QC procedures were performed to test naringin and naringenin stability in the feces. These samples were maintained at 37°C for 12 h. The detected naringin and naringenin amount was less than 20% of the amount added at 37°C over night, indicating poor cumulative excretion due to significant metabolism by gut enzymes.

**Table 8:** The cumulative excretion percentage of naringin and naringenin in human excreta (*n*=8 per groups, mean ± SD).

Dose (mg)	In urine (% of dose)			In feces (% of dose)			In urine and feces (% of dose)
	Naringin	Naringenin	Total	Naringin	Naringenin	Total	
40	0.067 ± 0.039	6.69 ± 6.20	6.77 ± 6.20	0.122 ± 0.202	0.259 ± 0.734	0.305 ± 0.857	7.08 ± 7.06
80	0.037 ± 0.013	8.82 ± 5.46	8.87 ± 5.46	0.023 ± 0.036	0.034 ± 0.080	0.048 ± 0.109	8.92 ± 5.57
160	0.027 ± 0.014	9.67 ± 6.07	9.70 ± 6.08	0.016 ± 0.023	0.053 ± 0.150	0.092 ± 0.196	9.79 ± 6.28
160 (high-fat diet)	0.025 ± 0.008	9.50 ± 5.00	9.53 ± 5.00	9.85 ± 20.0	1.23 ± 2.54	10.7 ± 22.3	20.2 ± 27.3
320	0.033 ± 0.026	15.7 ± 8.73	15.7 ± 8.73	0.126 ± 0.325	0.592 ± 1.41	0.717 ± 1.74	16.4 ± 10.5
480	0.012 ± 0.004	10.4 ± 8.43	10.4 ± 8.43	0.023 ± 0.033	0.799 ± 1.92	0.821 ± 1.95	11.2 ± 10.4
160, three times a day	0.034 ± 0.014	28.3 ± 43.2	28.4 ± 43.2	0.563 ± 1.00	1.83 ± 2.32	2.36 ± 3.07	30.7 ± 46.2

## Discussion and Conclusions

### Bioanalytical Methods

Initially, naringin-D4 and naringenin-D4 were selected to use as IS's for the analysis of naringin and naringenin in rat and dog plasma. However, during the pre-test of the nonclinical samples, naringin-D4 and naringenin-D4 showed significant levels of naringin and naringenin impurities, which prevented these compounds to use them as IS's. Instead, the structural analog quercetin 3-ß-D-glucoside was selected as an IS for the rat and dog plasma samples. In the clinical samples, quercetin 3-ß-D-glucoside IS showed an interference with 3-ß-D-glucoside in blank human plasma, therefore, quercetin 3-ß-D-glucoside could not be used as an IS in the human plasma samples. However, purified batches of naringin-D4 and naringenin-D4 became available with no naringin and naringenin impurities, therefore, naringin-D4 and naringenin-D4 were used as IS's in the clinical samples. Based on methods available in the literature in rats (Tsai and Tsai, 2012; Tong et al., 2012; Wen et al., 2012; Li S. Q. et al., 2013; Jin et al., 2015; Lu et al., 2015; Zeng et al., 2018) and humans (Ishii et al., 2000; Xiong et al., 2014), separate bioanalytical methods were used for the analysis of naringin and naringenin in rat, dog, and human samples. Compared to other methods available in the literature on human plasma and urine analysis (Ishii et al., 2000; Xiong et al., 2014), naringin-D4 and naringenin-D4 were used as IS's in this study as they were found to have less inference with the endogenous components of the human samples. Furthermore, these bioanalytical methods proved to be more reliable and robust for the quantitation of naringin and naringenin, which are important for the successful completion of nonclinical and clinical studies.

### Pharmacokinetic Characteristics and Species Differences

**Tables 1** and **2** demonstrated that, compared to nonclinical species, naringin pharmacokinetic processes were prolonged in humans owing to physiologic variables such as blood rate, biochemical processes, and clearance, which are related to the weight or body surface area of animal species (including humans) (Leon and Yu, 2016). Although similar to naringin, the naringenin absorption processes were affected by the size of the experimental animal; naringenin *t_1/2_* values for nonclinical species were close to that of humans for unknown reasons. In addition, in these animals, the AUC for naringenin administered by p.o. administration was markedly greater than that observed for i.v. administration, demonstrating prominent biotransformation of naringin into naringenin by intestinal microflora (Chen et al., 2018). After single-dose administration to rats, dogs, and humans, the pharmacokinetic behavior of naringenin followed linear pharmacokinetics, whereas the relationship between dose and AUC appeared nonlinear for naringin. Naringin is likely a precursor of various metabolites, maintaining a slightly fluctuating and increasing AUC for naringin and a linearly increased AUC for naringenin. The AUC for naringenin increased more rapidly with the dose in rats and humans than in dogs where the observed increase was slower. For naringenin in rats and humans, continued dose increase might saturate the enzyme pathways for its metabolism. Thus, the increase in AUC may be higher than expected as a smaller amount of naringenin is being eliminated (i.e., more naringenin is retained). Conversely, naringenin absorption in dogs could become saturated, resulting in lower than expected changes in AUC. The absolute bioavailability of naringin was 44.1% for rats and 34.4% for dogs, indicating a substantial first-pass effect of naringin in these animals. Moreover, compared with the report in aged rats (Zeng et al., 2019), the pharmacokinetic parameters of females were similarly significantly higher than those of males in rats and humans that was likely due to gender-related differences of CYP (Scandlyn et al., 2008).

### Metabolism and Species Differences

Naringin metabolites have been reported in urine, feces, and intestinal microbes of rats, dogs, and humans (Liu M. et al., 2012; Zeng et al., 2017; Chen et al., 2018), indicating species difference in metabolism. Utilising phase I biotransformation reaction pathways, naringin was metabolised to naringenin and their hydroxylated, hydrogenated, and dehydrogenated metabolites in rats and dogs, whereas small amounts of neoeriocitrin were detected in human urine and feces. The common glucuronidation and sulphate conjugation (phase II) reactions were observed in rats, dogs, and humans, while methylation reactions only occurred in rats and dogs. In addition, for some fission metabolites such as 2, 4, 6-trihydroxybenzoic acid, 4-hydroxybenzoic acid, hippuric acid, and 4-hydroxyhippuric acid, only trace amounts could be detected in human urine and fecal samples.

Our study focused on naringin and naringenin metabolites in liver and kidney microsomes and their CYP enzyme pathways, which could contribute to learning more about the metabolic processes *in vivo*, as well as species differences. With the exception of the two methylated metabolites in rats, naringin and naringenin were metabolised to the same ten metabolites, indicating a slight metabolic difference between rat and human liver microsomes. Furthermore, a blank solution or five single inhibitor working solutions were added to the incubation system III-human to evaluate naringin and naringenin metabolic enzyme pathways. The results demonstrated that naringin metabolism was a complex process catalyzed simultaneously by multiple enzymes, and may not be influenced only by a single CYP inhibitor.

### Excretion and Species Differences

In previous naringin excretion studies in rats and dogs, Liu et al. reported cumulative excretion percentage of 21% for rats and 60% for dogs in the forms of naringin, naringenin, and 4-hydroxyphenylpropionic acid (Liu M. et al., 2012); Zeng et al. presented a cumulative excretion percentage of 19% in the forms of naringin and naringenin, and 98% for aged rats in the forms of naringin, naringenin, and 4-hydroxyphenylpropionic acid (Zeng et al., 2019). In our clinical studies, the cumulative excretion percentage of the dose was less than 20% in the forms of naringin and naringenin. In mass balance studies, 4-hydroxyphenylpropionic acid amount was excluded owing to the unrealistic value, above 100%, obtained in humans. The observation was attributed to 4-hydroxyphenylpropionic acid metabolism from other flavonoids present in various foods such as vegetables, fruits, and beans. In studies on human intestinal microbes, Chen et al. reported the metabolism of isotope-labeled naringin, which can be used to explain the entire dose of naringin administered to subjects (Chen et al., 2018). Naringin and naringenin were excreted within 24 h in rats and dogs, and 48 h in humans, demonstrating a prolonged excretion process in humans. Interspecies differences can be attributed to physiological variables such as blood rate, clearance, biochemical processes, and variable intestinal motility, which are, in turn, related to the weight or body surface area of different species (Leon and Yu, 2016).

In summary, the pharmacokinetics and metabolism of naringin were studied in nonclinical and clinical trials. The pharmacokinetic parameters of naringin and its active metabolite naringenin were estimated through single-dose (p.o. and i.v.) and multiple-dose studies in rats, dogs, and humans. Twelve metabolites were identified with the incubation with rat and human liver microsomes and the metabolism was demonstrated as a complex and multipathway process in human liver microsomes. Species differences were indicated in the pharmacokinetics and metabolism of naringin. Moreover, naringin data can be employed to design clinical dosage regimens, enhance knowledge on underlying mechanisms, and promote novel drug development.

## Data Availability Statement

All datasets generated for this study are included in the article/**Supplementary Material**.

## Ethics Statement

The studies involving human participants were reviewed and approved by Ethics committees of Beijing Hospital; Beijing Hospital. The patients/participants provided their written informed consent to participate in this study. The animal study was reviewed and approved by Animal Ethics Committee of the School of Life Sciences in Sun Yat-sen University; Sun Yat-sen University.

## Author Contributions

YB and WS conceived and designed the experiments. YB, WP, CY, WZ, ML, LF, HW, and XZ performed the experiments. YB and WP analyzed the data. YB, PL, and WS wrote the paper.

## Funding

This work was financially supported by the National Natural Science Foundation of China (Grant Nos. 81173475 and 31571830), the National Major Scientific and Technical Special Project of China (Grant No. 2015ZX09101014), and the Science and Technology Planning Project of Guangdong Province of China (Grant No. 2015B020234004).

## Conflict of Interest

The authors declare that the research was conducted in the absence of any commercial or financial relationships that could be construed as a potential conflict of interest.

## Abbreviations

AUC, Area under the curve; CAD, Collisionally activated dissociation; CE, Collision energy; _Cmax_, Peak plasma level; CL, Apparent clearance; CYP, Cytochrome P450; CFR, Code of Federal Regulations; CXP, Collision cell exit potential; DP, Declustering potential; EP, Entrance potential; GCP, Good Clinical Practice ; HIV, human immunodeficiency virus; HQC, High quality control; ICH, International Council for Harmonization of Technical Requirements for Pharmaceuticals for Human Use; IS, Internal standard; LLOQ, Lower limit of quantification; LQC, Low quality control; MRT, Mean residence time; MQC, Middle quality control; MS/MS, Triple quadrupole mass spectrometry; QC, Quality control; Q-TOF-MS, Quadrupole time-of-flight mass spectrometry; SAS, Statistical Analysis System; SD, Standard deviation; SE, Standard error; *t*1/2, Terminal elimination half-life; _Tmax_, Time for peak plasma level; UHPLC, Ultra high-performance liquid chromatography; UHPLC/MS/MS, Ultra high-performance liquid chromatography coupled with triple quadrupole mass spectrometry; ULOQ, Upper limit of quantification; UPLC, Ultra performance liquid chromatography; UPLC/Q-TOF-MS, Ultra performance liquid chromatography coupled with quadrupole time-of-flight mass spectrometry; Vd, Apparent volume of distribution.
