# Supplementary material for: Pharmacokinetics and Metabolism of Naringin and Active Metabolite Naringenin in Rats, Dogs, Humans, and the Differences Between Species
Source: Front Pharmacol. 2020 Mar 27;11:364. doi: 10.3389/fphar.2020.00364 (PMC7118210; doi:10.3389/fphar.2020.00364)
Supplement: Supplementary file 1 [file DataSheet_1.docx]

Supplementary Material

**Journal name**

Frontiers in Pharmacology

**Article title**

Pharmacokinetics and metabolism of naringin and active metabolite naringenin in rats, dogs, humans and the differences between species.

**Contents**

Figure S1 (Page 15)

Figure S2 (Page 15)

Table S1 (Page 8)

Table S2 (Page 8)

Table S3 (Page 13)

Table S4 (Page 13)

Table S5 (Page 15)

**
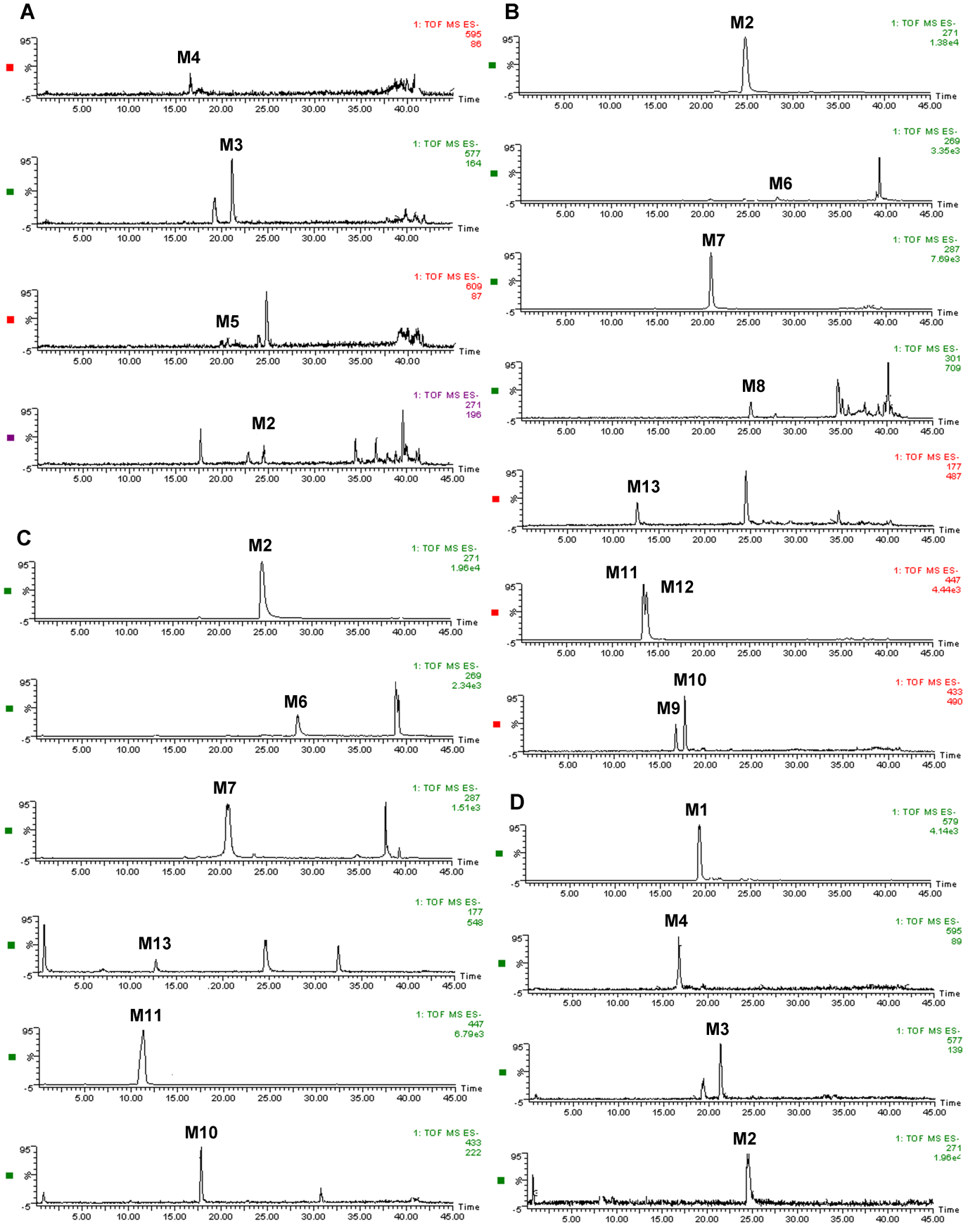
**

Figure S1. The extracted ion chromatograms of metabolites of naringin and naringenin in liver microsome.

**(A)** the metabolites of naringin in rat liver microsomes (system I-rat and II-rat); **(B)** the metabolites of naringenin in rat liver microsomes (system I-rat and II-rat); **(C)** the metabolites of naringenin in human liver microsomes (system I-human and II-human); **(D)** the metabolites of naringin in human liver microsomes (system I-human and II-human). M1: naringin, M2: naringenin, M3: rhoifolin, M4: neoeriocitrin, M5: hesperidin, M6: apigenin, M7: Eriodictyol, M8: Hesperetin, M9: Naringenin-O-glucoside, M10: Naringenin-O-glucoside, M11: Naringenin-O-glucuronide, M12: Naringenin-O-glucuronide, M13: 5,7-Dihydroxychromone.


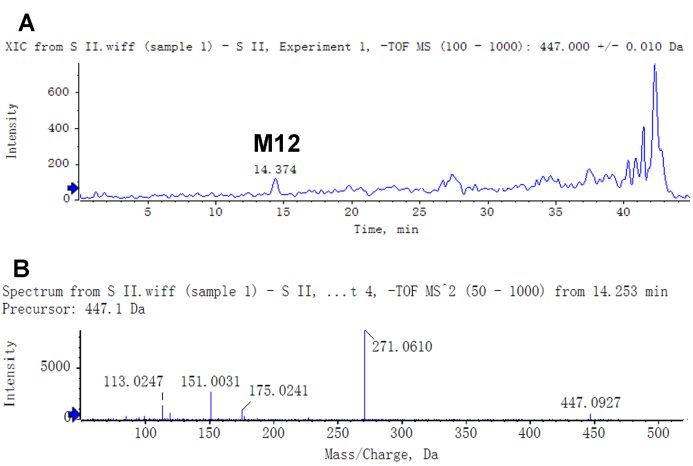


Figure S2. The extracted ion chromatograms of metabolites of naringin and naringenin in rat kidney microsome (system III-rat).

**(A)** extracted ion chromatograms of naringenin-O-glucuronide (M12); **(B)** the mass spectrum of M12. M12: Naringenin-O-glucuronide.

Table S1. The concentrations of calibration curves for naringin and naringenin in rats, dogs, and humans.

| Species | Biological  matrix | Analyte | Concentrations of calibration curve (ng/mL) | | | | | | | | |
| --- | --- | --- | --- | --- | --- | --- | --- | --- | --- | --- | --- |
|  |  |  | 1 | 2 | 3 | 4 | 5 | 6 | 7 | 8 | 9 |
| Rat |  |  |  |  |  |  |  |  |  |  |  |
|  | Plasma | Naringin | 4.89 | 9.78 | 1.96×10 | 3.91×10 | 9.78×10 | 1.96×10^2^ | 4.89×10^2^ | 9.78×10^2^ |  |
|  | Plasma | Naringenin | 2.04 | 4.07 | 1.02×10 | 2.04×10 | 1.02×10^2^ | 2.04×10^2^ | 5.09×10^2^ | 1.02×10^3^ |  |
| Dog |  |  |  |  |  |  |  |  |  |  |  |
|  | Plasma | Naringin | 2.04 | 5.09 | 1.02×10 | 2.04×10 | 5.09×10 | 1.02×10^2^ | 5.09×10^2^ | 1.02×10^3^ |  |
|  | Plasma | Naringenin | 2.04 | 5.10 | 1.02×10 | 2.04×10 | 5.10×10 | 1.02×10^2^ | 5.10×10^2^ | 1.02×10^3^ |  |
| Human |  |  |  |  |  |  |  |  |  |  |  |
|  | Plasma | Naringin | 5.00×10^-1^ | 1.00 | 2.00 | 5.00 | 1.00×10 | 2.00×10 | 5.00×10 | 1.00×10^2^ | 2.00×10^2^ |
|  | Plasma | Naringenin | 2.00 | 4.00 | 8.00 | 2.00×10 | 4.00×10 | 8.00×10 | 1.00×10^2^ | 1.50×10^2^ | 2.00×10^2^ |
| Human |  |  |  |  |  |  |  |  |  |  |  |
|  | Urine | Naringin | 1.00×10 | 2.50×10 | 5.00×10 | 1.20×10^2^ | 2.50×10^2^ | 5.00×10^2^ | 8.00×10^2^ | 1.20×10^3^ | 2.00×10^3^ |
|  | Urine | Naringenin | 1.00×10^2^ | 2.50×10^2^ | 5.00×10^2^ | 1.20×10^3^ | 2.50×10^3^ | 5.00×10^3^ | 8.00×10^3^ | 1.20×10^4^ | 1.60×10^4^ |
| Human |  |  |  |  |  |  |  |  |  |  |  |
|  | Faeces | Naringin | 1.00 | 2.50 | 5.00 | 1.20×10 | 25.0×10 | 5.00×10 | 8.00×10 | 1.20×10^2^ | 2.00×10^2^ |
|  | Faeces | Naringenin | 1.00×10 | 2.50×10 | 5.00×10 | 1.20×10^2^ | 2.50×10^2^ | 5.00×10^2^ | 8.00×10^2^ | 1.20×10^3^ | 1.60×10^3^ |

Table S2. The concentrations of quality control samples (QCs) for naringin and naringenin in rats, dogs, and humans..

| Species | Biological  matrix | Analyte | Concentrations of QCs ^a^ (ng/mL) | | | |
| --- | --- | --- | --- | --- | --- | --- |
|  |  |  | LLOQ | LQC | MQC | HQC |
| Rat |  |  |  |  |  |  |
|  | Plasma | Naringin | 4.89 | 1.47×10 | 7.82×10 | 7.82×10^2^ |
|  | Plasma | Naringenin | 2.04 | 6.42 | 8.14×10 | 8.14×10^2^ |
| Dog |  |  |  |  |  |  |
|  | Plasma | Naringin | 2.04 | 6.12 | 8.14×10 | 8.14×10^2^ |
|  | Plasma | Naringenin | 2.04 | 6.12 | 8.16×10 | 8.16×10^2^ |
| Human |  |  |  |  |  |  |
|  | Plasma | Naringin | 5.00×10^-1^ | 1.50 | 4.50×10 | 1.60×10^2^ |
|  | Plasma | Naringenin | 2.00 | 6.00 | 4.50×10 | 1.60×10^2^ |
| Human |  |  |  |  |  |  |
|  | Urine | Naringin | 1.00×10 | 3.00×10 | 6.00×10^2^ | 1.60×10^3^ |
|  | Urine | Naringenin | 1.00×10^2^ | 3.00×10^2^ | 6.00×10^3^ | 1.28×10^4^ |
| Human |  |  |  |  |  |  |
|  | Faeces | Naringin | 1.00 | 3.00 | 6.00×10 | 1.60×10^2^ |
|  | Faeces | Naringenin | 1.00×10 | 3.00×10 | 6.00×10^2^ | 1.28×10^3^ |

^a^ Three concentrations of QCs were shown as follows: one within three folds the lower limit of quantification (LLOQ) (LQC), one near the center (MQC), and one near the upper boundary of the standard curve (HQC)

Table S3. Results of bioanalytical method validation for naringin and naringenin in rats, dogs, and humans.

| Species | Biological  matrix | Analyte | Concentration  range (ng/mL) |  | Calibration curve  equation | R^2^ | Accuracy of LLOQ  and QCs ^a^ (%) | | Precision of LLOQ  and QCs ^a^ (%) | |
| --- | --- | --- | --- | --- | --- | --- | --- | --- | --- | --- |
|  |  |  |  |  |  |  | Within-run | Between-run | Within-run | Between-run |
| Rat |  |  |  |  |  |  |  |  |  |  |
|  | Plasma | Naringin | 4.89–9.78×10^2^ |  | y=1.22x+0.0362 | 0.999 | 90.5~111 | 96.7~102 | 1.99~13.0 | 5.63~10.8 |
|  | Plasma | Naringenin | 2.04–1.02×10^3^ |  | y=1.06x+0.00721 | 0.992 | 88.2~113 | 96.6~101 | 1.12~ 7.17 | 3.93~13.3 |
| Dog |  |  |  |  |  |  |  |  |  |  |
|  | Plasma | Naringin | 2.04–1.02×10^3^ |  | y=0.730x+0.00171 | 0.995 | 94.4~106 | 96.3~105 | 2.39~3.91 | 4.48~8.42 |
|  | Plasma | Naringenin | 2.04–1.02×10^3^ |  | y=0.846x+0.00312 | 0.993 | 91.1~105 | 93.7~ 99.0 | 0.913~7.75 | 3.07~6.94 |
| Human |  |  |  |  |  |  |  |  |  |  |
|  | Plasma | Naringin | 5.00×10^-1^–2.00×10^2^ |  | y=1.29x+0.00224 | 0.995 | 88.7~107 | 97.2~ 98.1 | 2.11~12.3 | 5.34~9.29 |
|  | Plasma | Naringenin | 2.00–2.00×10^2^ |  | y=2.55x+0.139 | 0.998 | 87.5~109 | 97.0~101 | 1.47~ 7.45 | 5.56~6.59 |
| Human |  |  |  |  |  |  |  |  |  |  |
|  | Urine | Naringin | 1.00×10–2.00×10^3^ |  | y=1.27x-0.000164 | 0.996 | 88.6~106 | 95.5~98.8 | 1.66~8.34 | 4.52~ 6.29 |
|  | Urine | Naringenin | 1.00×10^2^–1.60×10^4^ |  | y=0.932x+0.0413 | 0.997 | 84.1~115 | 96.3~98.3 | 1.21~5.79 | 3.38~10.5 |
| Human |  |  |  |  |  |  |  |  |  |  |
|  | Faeces | Naringin | 1.00–2.00×10^2^ |  | y=1.25x+0.000651 | 0.997 | 90.1~110 | 100~107 | 1.61~14.0 | 2.05~14.9 |
|  | Faeces | Naringenin | 1.00×10–1.60×10^3^ |  | y=0.801x+0.0332 | 0.995 | 99.4~113 | 102~109 | 1.31~15.6 | 2.27~ 9.44 |

^a^ Three concentrations of QCs (*n*=18) were in each run as follows: LQC, MQC, and HQC.

Table S4. Results of bioanalytical method validation for naringin and naringenin in rats, dogs, and humans (recovery and matrix effect).

| Species | Biological  matrix | Analyte | Coefficient of variation (%) | | | | | |
| --- | --- | --- | --- | --- | --- | --- | --- | --- |
|  |  |  | Recovery ^a^ | | |  | Matrix factors ^b^ | |
|  |  |  | LQC | MQC | HQC |  | LQC | HQC |
| Rat |  |  |  |  |  |  |  |  |
|  | Plasma | Naringin | 7.77 | 6.20 | 4.37 |  | 8.23 | 2.29 |
|  | Plasma | Naringenin | 3.69 | 5.82 | 3.04 |  | 4.12 | 2.80 |
| Dog |  |  |  |  |  |  |  |  |
|  | Plasma | Naringin | 1.33 | 2.09 | 2.81 |  | 5.31 | 5.90 |
|  | Plasma | Naringenin | 7.04 | 2.38 | 2.22 |  | 4.05 | 4.52 |
| Human |  |  |  |  |  |  |  |  |
|  | Plasma | Naringin | 10.1 | 3.50 | 4.13 |  | 2.56 | 1.54 |
|  | Plasma | Naringenin | 3.44 | 4.57 | 3.33 |  | 1.09 | 1.21 |
| Human |  |  |  |  |  |  |  |  |
|  | Urine | Naringin | 9.96 | 3.69 | 4.72 |  | 1.25 | 1.59 |
|  | Urine | Naringenin | 9.84 | 4.78 | 2.96 |  | 3.72 | 0.330 |
| Human |  |  |  |  |  |  |  |  |
|  | Faeces | Naringin | 2.30 | 1.49 | 1.54 |  | 1.83 | 1.16 |
|  | Faeces | Naringenin | 3.77 | 0.984 | 1.21 |  | 2.11 | 1.84 |

^a^ Three concentrations of QCs (*n*=18) were in recovery experiments as follows: LQC, MQC, and HQC.

^b^ The matrix effect experiments were performed using three determinations per concentration at two concentrations from at least six sources of blank matrices (LQC, *n*=18; HQC, *n*=18).

Table S5. Identification of metabolites of naringin and naringein in liver and kidney microsomes of rats and humans by LC-MS

| No. | Name | Formula | [M-H]^-^  (Error, ppm) | Main fragment ions (*m/z*) |
| --- | --- | --- | --- | --- |
| M1 | Naringin | C_27_H_32_O_14_ | 579.1699 (-2.6) | 459 [M-H-C_8_H_8_O]^-^, 271 [M-H-Rha-Glu]^-^, 151 [M-H-Rha-Glc-C_8_H_8_O]^-^ |
| M2 | Naringenin | C_15_H_12_O_5_ | 271.0581 (-9.2) | 177[M-H-C_6_H_6_O]^-^, 151 [M-H-C_8_H_8_O]^-^, 119[M-H-C_7_H_4_O_4_]^-^ |
| M3 | Rhoifolin | C_27_H_30_O_14_ | 577.1561 (0.7) | 459 [M-H-C_8_H_6_O]^-^, 269 [M-H-Rha-Glu]^-^, 151[M-H-Rha-Glc-C_8_H_6_O]^-^ |
| M4 | Neoeriocitrin | C_27_H_32_O_15_ | 595.1653 (-1.7) | 459[M-H-C_8_H_8_O_2_]^-^, 287[M-H-Rha-Glu]^-^, 151 [M-H-Rha-Glu-C_8_H_8_O_2_]^-^ |
| M5 | Hesperidin | C_28_H_34_O_15_ | 609.1791 (-4.6) | 459[M-H- C_9_H_10_O_2_]^-^, 271[M-H-CH_3_O-Rha-Glu]^-^, 151 [M-H -Rha-Glu-C_9_H_10_O_2_]^-^ |
| M6 | Apigenin | C_15_H_10_O_5_ | 269.0468 (6.7) | 225 [M-H-CO_2_]^-^, 151 [M-H-C_8_H_6_O]^-^, 117 [M-H-C_7_H_4_O_4_]^-^ |
| M7 | Eriodictyol | C_15_H_12_O_6_ | 287.0561 (1.4) | 151 [M-H-C_8_H_8_O_2_]^-^, 135 [M-H- C_7_H_4_O_4_]^-^, 107 [M-H-C_8_H_8_O_2_-CO]^-^ |
| M8 | Hesperetin | C_16_H_14_O_6_ | 301.0736 (8.0) | 286[M-H-CH_3_]^-^, 177[M-H-C_6_H_4_O_3_]^-^, 151 [M-H-C_9_H_10_O_2_]^-^, 107 [M-H-C_9_H_10_O_2_-CO_2_]^-^ |
| M9 | Naringenin-O-glucoside | C_21_H_20_O_10_ | 433.1175 (9.2) | 271 [M-H-Glu]^-^, 151[M-H-Glu-C_8_H_8_O]^-^ |
| M10 | Naringenin-O-glucoside | C_21_H_20_O_10_ | 433.1157 (5.1) | 271 [M-H-Glu]^-^, 151[M-H-Glu-C_8_H_8_O]^-^ |
| M11 | Naringenin-O-glucuronide | C_21_H_20_O_11_ | 447.0897 (-6.7) | 271[M-H-GluA]^-^, 151 [M-H-GluA-C_8_H_8_O]^-^, |
| M12 | Naringenin-O-glucuronide | C_21_H_20_O_11_ | 447.0891 (-5.8) | 271[M-H-GluA]^-^, 151 [M-H-GluA-C_8_H_8_O]^-^, |
| M13 | 5,7-Dihydroxychromone | C_9_H_6_O_4_ | 177.0168 (-5.6) | 133 [M-H-CO_2_]^-^ |
